# Lipid Droplets: A Significant but Understudied Contributor of Host–Bacterial Interactions

**DOI:** 10.3390/cells8040354

**Published:** 2019-04-15

**Authors:** Cassandra L. Libbing, Adam R. McDevitt, Rea-Mae P. Azcueta, Ahila Ahila, Minal Mulye

**Affiliations:** Division of Biomedical Sciences, College of Osteopathic Medicine, Marian University, 318e Michael A. Evans Center for Health Sciences, 3200 Cold Spring Road, Indianapolis, IN 46222, USA; clibbing516@marian.edu (C.L.L.); amcdevitt589@marian.edu (A.R.M.); razcueta990@marian.edu (R.-M.P.A.); aahila489@marian.edu (A.A.)

**Keywords:** obligate intracellular bacteria, facultative intracellular bacteria, extracellular bacteria, lipid droplets, PGE2, microbiota

## Abstract

Lipid droplets (LDs) are cytosolic lipid storage organelles that are important for cellular lipid metabolism, energy homeostasis, cell signaling, and inflammation. Several bacterial, viral and protozoal pathogens exploit host LDs to promote infection, thus emphasizing the importance of LDs at the host–pathogen interface. In this review, we discuss the thus far reported relation between host LDs and bacterial pathogens including obligate and facultative intracellular bacteria, and extracellular bacteria. Although there is less evidence for a LD–extracellular bacterial interaction compared to interactions with intracellular bacteria, in this review, we attempt to compare the bacterial mechanisms that target LDs, the host signaling pathways involved and the utilization of LDs by these bacteria. Many intracellular bacteria employ unique mechanisms to target host LDs and potentially obtain nutrients and lipids for vacuolar biogenesis and/or immune evasion. However, extracellular bacteria utilize LDs to either promote host tissue damage or induce host death. We also identify several areas that require further investigation. Along with identifying LD interactions with bacteria besides the ones reported, the precise mechanisms of LD targeting and how LDs benefit pathogens should be explored for the bacteria discussed in the review. Elucidating LD–bacterial interactions promises critical insight into a novel host–pathogen interaction.

## 1. Introduction 

Lipid droplets (LDs) are lipid storage organelles comprising a phospholipid monolayer surrounding a hydrophobic core of neutral lipids: cholesterol esters (CEs) and triacylglycerols (TAGs). LD biogenesis occurs in the endoplasmic reticulum (ER) when the enzymes Acyl-CoA:cholesterol acyltransferase (ACAT) and Acyl-CoA:diacylglycerol acyltransferase (DGAT) esterify excess cholesterol and free fatty acids [1,2,3]. The newly formed LDs carry several proteins in the phospholipid monolayer including the perilipin (Plin) family and, lipid biosynthetic and lipolytic enzymes [4]. Plin1 and Plin2 are important LD coat proteins which regulate LD formation and breakdown [5]. During cell starvation, two lipolytic enzymes mediate LD breakdown: adipose triacylglycerol lipase (ATGL) converts TAG to diacylglycerol (DAG) [6], and hormone-sensitive lipase (HSL) converts DAG to monoacylglycerol (MAG) [7]. The enzyme phospholipase A (PLA), important for LD homeostasis, also hydrolyzes fatty acids. PLA2 particularly mobilizes arachidonic acids (AA) from the cellular phospholipid bilayer as well as the LD phospholipid monolayer to produce eicosanoid lipid immune mediators, such as leukotrienes, lipoxins, and prostaglandins [8]. LDs are important for many cellular processes including but not limited to lipid metabolism, membrane trafficking and cell signaling [9]. They are involved in the pathogenesis of metabolic diseases such as diabetes, obesity, atherosclerosis [10], as well as cancer development [11] and neurodegenerative diseases [12,13]. They also play an important role in leukocytic inflammatory responses [14,15] and are emerging as key players during host–pathogen interactions. Numerous studies in the past decade increasingly support the notion that various parasites, fungi, viruses and bacteria target host LDs to enhance both immune evasion and microbial proliferation [16,17,18,19]. Thus far, several reviews have comprehensively described the importance of LDs during host–pathogen interactions [16,17,18,20,21,22,23,24]. A recent review on parasite–LD interactions outlines that host and parasite LDs serve as a source of lipids and energy, maintain parasite homeostasis through heme detoxification and provide eicosanoids to promote immune evasion, thereby supporting parasite survival [24]. Various other reviews discuss the importance of host LDs for viral replication, assembly, and infection [16,25,26,27]. While several bacteria target host LDs to facilitate infection and survival, most reviews thus far predominantly focus on the role of LDs in intracellular pathogen *Mycobacterium species pluralis (spp.)* and *Chlamydia spp*. pathogenesis [16,17,18,22,28,29]. Since Rudolf Virchow’s description in 1863 of LD-laden foam cells in lepromatous lesion biopsies from *Mycobacterium leprae*-infected patients [28,30], many bacteria have been reported to interact with host LDs. However, their importance and specific roles in bacterial pathogenesis is still being elucidated. Our review comprehensively discusses the role of LDs during infection with (1) intracellular bacteria which reside within the host cell (*Chlamydia spp, Coxiella burnetii, Anaplasma phagocytophilum, Rickettsia spp., Mycobacterium spp*., *Salmonella spp.*) and, (2) extracellular bacteria which do not invade host cells and reside in the extracellular matrix (*Pseudomonas aeruginosa, Streptococcus pyogenes* and *Vibrio cholerae*) [31]. Although many bacteria share mechanisms to target host LDs, differences in their infectious cycle and lifestyle do allow for unique host LD–bacterial interactions. 

## 2. Intracellular Bacteria

Intracellular bacteria establish contact with a susceptible host and multiply within the cells [32], a strategy employed to escape the humoral immune components (complement proteins and antibodies), as well as antibiotics to facilitate survival [33]. Intracellular bacterial pathogens infect a wide range of host cells, professional phagocytes like macrophages and non-professional phagocytes including epithelial cells, endothelial cells, and hepatocytes [32,34]. Classically, intracellular pathogens are further categorized as facultative intracellular or obligate intracellular [32] based on their ability or inability, respectively, to multiply in a cell-free environment. Some examples of obligate intracellular bacteria include *Chlamydia spp.* and *Rickettsia spp.*, whereas *Francisella tularensis, Listeria monocytogenes, Salmonella spp.* and *Mycobacterium spp.* are examples of facultative intracellular bacteria [31,32]. In this section, we will discuss the currently reported LD–pathogen interactions of obligate and facultative intracellular bacteria while also assessing the mechanisms and physiologic role of LD manipulation during their distinct lifecycles. 

### 2.1. Obligate Intracellular Bacteria

Similar to viruses, obligate intracellular bacteria lack the ability to live outside the host cells. On host cell entry, each intracellular bacterium follows a different lifestyle; while some modify the phagosome to form a pathogen-containing vacuole (vacuolar pathogens, e.g., *Chlamydia spp., Coxiella burnetii* and *Legionella pneumophila*), some escape the vacuole and multiply in the cytoplasm (cytoplasmic pathogens, e.g., *Listeria monocytogenes*, *Shigella flexneri, Burkholderia spp, Rickettsia spp.* etc.) [35]. Despite differences in multiplication sites, these bacteria depend on the host cell for energy and nutrients owing to the lack of many biosynthetic pathways and inability to perform energy metabolism. Many intracellular bacteria manipulate host cell lipids for entry, avoidance of phagosome formation, vacuolar biogenesis, and intracellular multiplication [23,36]. As LDs are lipid storage organelles and a crucial component of cellular lipid metabolism, understanding the role of LDs in vacuolar and cytoplasmic intracellular bacterial pathogenesis is imperative. 

#### 2.1.1. *Chlamydia spp.*

The *Chlamydia spp.* are a group of Gram-negative, obligate intracellular bacteria that cause a wide range of acute and chronic diseases such as trachoma, conjunctivitis, pelvic inflammatory disease (*C. trachomatis)* and atypical pneumonias (*C. psittaci, C. pneumoniae)*. While *C. psittaci* can infect humans as well as birds, *C. trachomatis* and *C. pneumoniae* are primarily human pathogens. Despite varied disease presentations, all *Chlamydiae* share a similar life cycle wherein they alternate between two forms: the extracellular, infectious elementary body (EB) and the intracellular, replicative reticulate body (RB). The metabolically inert EBs infect the host mucosal cells, prevent phagolysosomal fusion, and establish a parasitic vacuole, also known as an inclusion; this inclusion is enclosed by a phospholipid bilayer creating a niche for *Chlamydial* intracellular survival. In the inclusion, EBs differentiate into RBs and, after several rounds of replication, RBs re-differentiate into EBs before being released from the host cell to infect neighboring cells. *Chlamydia spp.* lack several metabolic and biosynthetic pathways, resulting in their host cell dependence for the intermediates necessary for intracellular growth. To obtain host nutrients, the bacterium translocates effector proteins across the inclusion membrane into the host cytosol via its type 3 secretion system (T3SS); these effector proteins manipulate host cell processes, thereby obtaining host amino acids, iron, and lipids. More specifically, for inclusion membrane biogenesis, *Chlamydia spp.* intercept both host endocytic vesicles as well as secretory membrane trafficking vesicles [37,38]. *C. trachomatis* and *C. pneumoniae* also manipulate host LDs to promote infection; however, there are significant differences in *Chlamydiae*–LD interaction between species. Hence, we will discuss *C. trachomatis* and *C. pneumoniae* separately. 

##### *C.* *trachomatis*

Depending on the serovar, *C. trachomatis* causes a wide range of diseases which, all together, carry a remarkably large disease burden worldwide. Serovars Ab, B, Ba, C (trachoma biovar) cause trachoma, which is the leading cause of non-congenital blindness worldwide. Conversely, serovars D–K (genital tract biovar) cause the most prevalent bacterial sexually transmitted infection in the United States and, lastly, the lymphogranuloma venereum biovar causes invasive urogenital and anorectal infections. Although these infections can be treated with antibiotics, there is still a lack of cost-effective treatments and efficacious vaccines, thus emphasizing the need for better understanding of *C. trachomatis* pathogenesis. Due to its intracellular nature, it is clear that *C. trachomatis* manipulates host lipid metabolism. Several studies suggested that *C. trachomatis* induces host LD accumulation, supported not only by direct microscopic visualization but also increased CE levels in infected HeLa epithelial cells and mouse embryonic fibroblasts (MEFs) [39,40,41]. LDs associated with the inclusion membrane during early stages of infection, eventually translocating into the inclusion, and situating themselves between the inclusion membrane and the bacterial outer membrane [42]. Additionally, during murine infection with *C. muridarum,* a model organism used to study human *C. trachomatis* infections, LDs translocated into the inclusion, suggesting this phenomenon is not restricted to the human pathogen [43]. The distinct localization of the host LDs inside the inclusion suggests that, rather than assisting inclusion membrane biogenesis, host LDs potentially provide nutrients and lipids to the replicating bacteria [39,42]. 

*C. trachomatis* employs several proteins to intercept and utilize LDs as a substrate during bacterial multiplication. IncA, a *Chlamydial* inclusion membrane protein important for homotypic vesicle fusion [44], marked the inclusion membrane segments in HeLa epithelial cells and remained transiently associated with LDs, suggesting its role in LD translocation [42]. Although the role of LD-associated *Chlamydial* proteins, Lda1 and Lda2, is unknown [39], overexpression of Lda3 in *C. trachomatis*-infected HeLa cells decreased the expression of *plin2*, a gene coding for a LD coat protein; this LD “uncloaking” may assist in LD lipolysis and lipid availability. Ectopically expressed Lda3 sequentially colocalized to the cytoplasmic side of the inclusion, host LDs, and, finally, within the inclusion, suggesting Lda3 also plays a role in translocating LDs through the inclusion membrane [42]. Although IncA and ectopically expressed Lda3 are proposed to colocalize and assist in LD translocation, a proteomic study in *C. trachomatis*-infected HeLa cells demonstrated absence of endogenous IncA, Lda1, and Lda3 colocalization with LDs [40]. Further, only ectopically expressed Lda1 and Lda3 were detected on LDs [40], thus questioning the importance of the LD-associated proteins in LD translocation to the bacterial inclusion. Although the role of Lda3 and IncA remains unclear, the proteomic analysis highlighted that *C. trachomatis* enriches the LD proteome with host lipid metabolism and biosynthesis-related proteins. Additionally, the study demonstrated distinct host LD colocalization with bacterial inclusion proteins Cap1 (CT529), CTL0882 (CT618), and IncG (CT118) [40]. Although these findings support a strong interaction between *C. trachomatis* and host LDs, a thorough identification of the specific proteins involved in LD translocation, their role and at what stage post-infection they are important requires further investigation. 

While it is clear that *C. trachomatis* actively manipulates host LDs, their contribution to *C. trachomatis* growth is a topic of ongoing debate. In HeLa cells, pharmaceutical inhibition of LD formation enzymes fatty acyl-CoA synthetase [39,45] and ACAT [46] decreased bacterial growth. Notably, the inhibitor triacsin C used in these studies not only blocked host fatty acyl-CoA synthetase but also *Chlamydial* acyl-ACP (acyl carrier protein) synthase [47], indicating that growth inhibition could be an off-target effect. However, infection of DGAT1/DGAT2 double knockout mouse embryonic fibroblasts (MEFs) which lack the ability to form TAG-rich LDs demonstrated significantly reduced growth when compared to wild-type MEFs [40]. Several other studies also reported a partial reduction in bacterial growth with LD inhibition [42,46,48], emphasizing that LDs are not absolutely essential but do allow for optimal C. *trachomatis* growth. Supporting this claim, MEFs devoid of LDs, showed delayed inclusion and infectious progeny expansion, lagging behind their wild-type counterparts by 24 h [41], indicating that LDs support intracellular growth especially during the early phases of infection. In fact, LDs could be the first lipid source for metabolic processes required during replication. Several bacterial and host proteins are hypothesized to participate in this LD-mediated metabolic boost. *C. trachomatis* expresses a putative cholesterol esterase, CT149, which may hydrolyze CEs and TAGs, freeing cholesterol and free fatty acids from the LDs for bacterial use [49]. Further, intimate LD-RB association in HeLa cells suggested LDs as a source of phospholipids such as phosphatidylcholine (PC) and lyso-PC for the replicating bacterial progeny [42]. A host ER bound PC reacylation enzyme acyl-CoA:lysoPC acyltransferase 1 (hLPCAT) localized to the lipid network surrounding the inclusion during *C. trachomatis* infection, but did not enter the inclusion, suggesting its importance in modifying host PC to contain branched chain fatty acids for bacterial assimilation and support the expansion and lipid remodeling of the inclusion membrane [48]. Similarly, CT775, a membrane bound chlamydial lysophosphatidylcholine acyltransferase (cLPCAT) associated with LDs in oleic acid (OA), treated *C. trachomatis*-infected HeLa cells [50]. However, the interaction between cLPCAT and hLPCAT remains unknown. Interestingly, another host protein, human acyl-CoA carrier 6 (hACBD6) associated with LDs and its expression was directly proportional to cLPCAT, suggesting a synergistic activity of the host and the bacterial protein to potentially facilitate acquisition of membrane lipids [50]. Two other *Chlamydial* proteins, phosphatidyl serine decarboxylase CT699 and acyl-ACP synthase CT776, also putatively conferred *C. trachomatis* the ability to utilize host lipids [47]. Thus, *C. trachomatis* employs multiple strategies to use LD-derived lipids to promote faster replication and larger inclusions at least during the first 24 h post-infection [41]. 

In addition to directly providing lipids as a metabolic resource, LDs in *C. trachomatis*-infected cells could potentially deliver the host TAG lipase ATGL to mediate LD lipolysis which may aid bacterium pathogenicity [42]. Free fatty acids, such as arachidonic acids, released during LD lipolysis are a source of lipid immune mediators, including prostaglandin E2 (PGE2). Notably, *C. trachomatis* infection of HeLa cells upregulated PGE2 synthesis enzyme cyclooxygenase-2 (*cox-2*) expression as well as PGE2 protein levels, which consequently induced mononuclear leukocyte accumulation and host tissue damage [51]. Thus, LDs potentially contribute to the disease exacerbation during *C. trachomatis* infection. However, this process remains unexplored. 

##### *C.* *pneumoniae*

Although it primarily causes pneumonia, *C. pneumoniae* infection also results in the chronic inflammatory disease atherosclerosis [52,53], characterized by abnormal cellular lipid build-up, leading to LD-containing foam cell formation within arteries of the host [54]. In *C. pneumoniae*-infected mice, carotid artery segments demonstrated the presence of bacteria-containing macrophage foam cells [55]. In vitro, the induction of LD-containing foam cells in infected murine and human macrophages [56,57,58], and adipocytes [59], indicated that LDs play an important role in *C. pneumoniae* pathogenesis. In the low-density lipoprotein (LDL)-treated human macrophage-like cell line, THP-1, *C. pneumoniae* infection increased the CE to total cholesterol (TC) ratio and subsequently LD accumulation to form foam cells [57,58]. This LD accumulation was dependent on the cholesterol esterification enzyme, ACAT, supported by the fact that ACAT inhibition reduced the CE/TC ratio in infected cells. This suggests that *C. pneumoniae* infection alters host lipid metabolism to facilitate LD accumulation and hence foam cell formation. Along with ACAT, the nuclear receptors, PPARα and PPARγ, seemed to play an important role in *C. pneumoniae*-induced foam cell formation [56,58]. Treatment of *C. pneumoniae*-infected cells with the PPARα and PPARγ agonists, fenofibrate and rosiglitazone, respectively, significantly inhibited foam cell formation, suggesting that the bacterium downregulates these nuclear receptors to facilitate bacterial intracellular survival. PPARα and PPARγ are important regulators of fatty acid and cholesterol metabolism [60,61,62] which, when activated, induce expression of an important cholesterol efflux regulatory protein, ATP-binding cassette transporter A1 (ABCA1), promoting macrophage cholesterol efflux [62,63]. This suggests that *C. pneumoniae* hinders PPARα and/or PPARγ expression in order to block cholesterol efflux, thereby inducing the abnormal lipid accumulation needed for the synthesis of LD-laden foam cells, a characteristic of atherogenesis. Additionally, *C. pneumoniae* -stimulated phosphorylation of mitogen-activated protein kinases (MAPK) including JNK1/2 and ERK1/2 [56] also contributed to LD accumulation and atherogenesis [64]. Thus, *C. pneumoniae* manipulates host cell lipid metabolism to induce LD-laden foam cell formation. 

Studies demonstrate that besides foam cell formation, LDs may bolster *C. pneumoniae* intracellular growth. In murine bone marrow-derived macrophages (BMM), *C. pneumoniae* exploited the host innate immune signaling NLRP3 inflammasome for intracellular growth. Infected BMMs from *nlrp3^−/−^* mice altered LD numbers, indicating that *C. pneumoniae* utilizes NLRP3 to acquire host lipids [65]. Further, *C. pneumoniae* also required host toll-like receptor-2 (TLR2) signaling to induce LD formation in macrophages [66], suggesting that the bacterium exploits host innate immune response to induce LD accumulation. However, the contribution of LDs to bacterial growth and pathogenesis is still unclear. In adipocytes, *C. pneumoniae* replication was dependent on the LD breakdown enzyme, HSL, and lipid transport protein fatty acid binding protein (FABP4) activity [59]. These proteins liberate host free fatty acids which undergo β-oxidation to generate ATP. Thus, *C. pneumoniae* potentially uses host LDs to generate energy and promote intracellular growth. 

*C. trachomatis* and *C. pneumoniae* employ different mechanisms to induce LD accumulation and utilize LDs as an energy and lipid source for optimal intracellular growth. Despite inducing LD accumulation, it is unclear why *C. trachomatis* lacks atherogenic ability similar to *C. pneumoniae*. Since PGE2 contributes significantly to *C. trachomatis* [51] as well as *C. pneumoniae* [67] pathogenesis, LD contribution to lipid immune mediator production merits further investigation. 

#### 2.1.2. *Coxiella burnetii*

*C. burnetii*, a Gram-negative obligate intracellular pathogen, is the causative agent of Q fever which presents as an acute debilitating flu-like illness or chronic endocarditis. The bacterium spreads via aerosol transmission and preferentially infects alveolar macrophages. After entering the host cell via phagocytosis, the bacterium directs the formation of a parasitophorous vacuole (PV), a lysosome-like compartment essential for *C. burnetii* growth [68]. The bacterial PV is highly fusogenic and expands through homotypic fusion with early and late endosomes, lysosomes, and autophagosomes; these organelles contribute membranes, proteins, and lipids for PV biogenesis [69]. *C. burnetii* employs its type 4 secretion system (T4SS) to release effector proteins into the host cell cytoplasm and manipulate cellular processes for successful PV formation and maturation [70]. For example, a *C. burnetii* T4SS effector, *Coxiella* vacuolar protein B (CvpB), binds host lipid phosphatidylinositol 3-phosphate PI(3)P to assist in membrane trafficking and homotypic fusion for PV biogenesis [71]. Further, the *C. burnetii* PV membrane is sterol-rich [72] and, despite coding for two unique eukaryote-like sterol reductase homologs [73,74], *C. burnetii* does not appear to synthesize the predominant sterol, cholesterol, and instead obtains cholesterol from the host cell. Interestingly, elevated PV cholesterol levels during the early stages of infection are bactericidal for *C. burnetii,* suggesting that the bacterium reduces PV cholesterol during early stages of infection and increases it later for PV maintenance [75]. As LDs store esterified cholesterol and fatty acids, they are an attractive source of lipids for *C. burnetii* intracellular growth. 

Several LD metabolism-related genes such as *plin2, acat* and *fabp4* were differentially regulated in *C. burnetii*-infected human [76,77] and mouse alveolar macrophages [78]. Intriguingly, similar to Virchow’s observation in leprosy patients, the heart valves of Q fever endocarditis patients showed the presence of *C. burnetii*-infected, LD-filled foam cells [79]. LDs were also observed in the bacterial PV lumen of infected human alveolar macrophages [80]. Additionally, in *C. burnetii*-infected mouse alveolar macrophages, increased LD numbers were dependent on the bacterial T4SS [78], indicating that *C. burnetii* actively manipulates host LDs. Moreover, alteration of host LD homeostasis affects bacterial intracellular growth. Treatment of infected monkey kidney epithelial cells with a broad spectrum antiviral molecule ST699, which localizes to host cell LDs, inhibited bacterial growth [81]. siRNA depletion of the LD breakdown enzyme, ATGL, increased the number of *C. burnetii* PVs in HeLa epithelial cells [78]; however, its effect on bacterial growth is not known. Exploring this further, infection of mouse alveolar macrophages demonstrated that blocking ATGL activity almost completely inhibited bacterial growth, indicating LD breakdown is essential for *C. burnetii* intracellular growth [78]. Conversely, blocking ACAT and DGAT-mediated LD formation increased bacterial growth. Thus, LD breakdown could potentially serve as a source of free fatty acids and cholesterol to support *C. burnetii* PV biogenesis. However, specifically inhibiting DGAT-mediated TAG formation abolished all LDs from *C. burnetii*-infected mouse alveolar macrophages. This suggests that the primary role of LDs during infection is TAG and not CE mobilization [78]. The ATGL-mediated breakdown of TAG-rich LDs releases fatty acids, suggesting that LD-derived lipids could serve as a nutrient source for *C. burnetii* instead of providing cholesterol for bacterial growth.

Recently, Stead et al. reported that *C. burnetii* phospholipase A (PldA), an enzyme which catalyzes the removal of an acyl chain from a phospholipid to produce free fatty acids, is important for bacterial intracellular growth [82]. In axenic media, when *C. burnetii* is in the non-replicating stationary small cell variant (SCV) phase, PldA assisted the accumulation of free fatty acids. These lipids potentially provide nutrients to the intracellular replicating logarithmic phase large cell variant (LCV) during initial stages of infection when the PV is limited in nutrients [82]. Additionally, similar to *C. trachomatis* and *C. pneumoniae*, PGE2 plays an important role during *C. burnetii* pathogenesis. PGE2 mediated immunosuppression in vitro in *C. burnetii*-infected cells and in vivo in patients [83,84,85] to promote bacterial intracellular growth. As LDs are a source of arachidonic acids, precursors for PGE2 synthesis, LD breakdown could result in PGE2-mediated immune modulation during *C. burnetii* infection. Thus, LDs could serve as a nutrient and/or immune modulator source to facilitate *C. burnetii* intracellular growth. However, the mechanism and the bacterial effector proteins employed to manipulate host LD homeostasis and the contribution of LDs to *C. burnetii* pathogenesis remains unknown. 

#### 2.1.3. *Anaplasma phagocytophilum*

*A. phagocytophilum,* a member of the *Anaplasmatacae* family, is a Gram-negative, pleiomorphic, obligate intracellular tick-borne pathogen that causes human granulocytic anaplasmosis [86,87]. It preferentially infects neutrophils, utilizes caveolae to enter the host cell, and then multiplies within vacuoles called inclusions or morulae [88]. *A. phagocytophilum* is an obligate intracellular pathogen for multiple reasons: first, the structural integrity of the inclusion membrane and therefore bacterial intracellular growth depends on host cell cholesterol level maintenance [88,89]. Additionally, due to the lack of typical cell wall constituents, lipid A and peptidoglycan, the bacterium itself depends on host cholesterol for cell wall structural integrity [88]. To make up for its lack of cholesterol biosynthesis genes [90], the bacterium essentially hijacks the host cell LDL trafficking and recruits cholesterol to the inclusion membranes [89]. While it is clear that *A. phagocytophilum* manipulates this host cell lipid delivery system to its advantage, specific mechanisms that it utilizes to recruit host cell cholesterol are yet to be elucidated. Recent studies demonstrate that *plin1* is essential for *A. phagocytophilum* infection and multiplication [91,92], suggesting LDs may play an important role in cholesterol recruitment. In infected promyelocytic human HL-60 cells, *plin1* mRNA and protein levels were directly proportional to bacterial multiplication and increased *plin1* expression was beneficial to the bacterium [91]. How the bacterium increased *plin1* levels is unclear, although *A. phagocytophilum* possesses a functional T4SS which secrets effectors known to modulate host cell autophagosome formation and SUMOylation [93,94]; whether these T4SS effectors modulate *plin1* expression requires further elucidation. Additionally, how increased Plin1 levels facilitate *A. phagocytophilum* growth is still unknown. One hypothesis posits that because Plin1 regulates LD lipolysis, change in its expression leads to host cell cholesterol and fatty acid release to support bacterial intracellular growth and survival [95,96]. Comparing relative changes in Plin and cholesterol levels in infected cells will address this hypothesis. Although LDs seem to be important for *A. phagocytophilum* infection, in order to establish a direct LD–pathogen relationship, future studies should quantify LD accumulation in infected cells. Additionally, the mechanisms *A. phagocytophilum* employs to target host LDs and their contribution to bacterial growth should be investigated. 

Interestingly, despite a similar intracellular lifestyle, no reports suggest LD interaction with *Ehrlichia chaffeensis,* a bacterium that also belongs to the *Anaplasmatacae* family [97]. *E. chaffeensis* causes human monocytic ehrlichiosis and, similar to *A. phagocytophilum,* depends on host cell cholesterol for intracellular survival [88]; this commonality highlights an opportunity for future studies to explore the role of LDs during *E. chaffeensis* infection. 

#### 2.1.4. *Orientia tsutsugamushi*

Unlike the obligate intracellular bacteria discussed thus far, *O. tsutsugamushi* resides in the host cell cytoplasm rather than a parasitophorous vacuole. This Gram-negative coccobacillus belongs to the genus *Rickettsia* and is transmitted to humans by arthropods [98], ultimately resulting in scrub typhus. In humans, *O. tsutsugamushi* spreads hematogenously, mainly infecting the endothelial cells [99] via a clathrin-mediated endocytic pathway [100]. Once endocytosed, the bacterium escapes the endosomal vacuole and replicates freely in the cytoplasm. It then buds in a viral-like process and egresses from the host cell [101,102]. Although *O. tsutsugamushi* departure from the cell requires host cell cholesterol [103], little is known about its lipid–pathogen interactions [21], with especially less evidence for LD involvement. Thus far, only one report demonstrated a time-dependent LD increase in *O. tsutsugamushi*-infected L929 fibroblasts [103]. Furthermore, there was a direct correlation between the size and number of host LDs and bacterial multiplication [103]. Based on cellular lipid analysis, TAG concentration increased in infected cells but cholesterol and phospholipid levels remained unchanged; this indicates that the LDs are TAG-rich [103]. Although the increase in cellular TAG suggested LDs as a potential energy and nutrient source similar to *Chlamydia spp.*-infected cells, their contribution to *O. tsutsugamushi* infection remains unknown. Elucidation of how LD accumulation affects bacterial growth or egress will clarify the importance of LDs during *O. tsutsugamushi* pathogenesis. 

Thus far, *O. tsutsugamushi* is the only member of the genus *Rickettsia* reported to demonstrate a LD–pathogen interaction. *R. prowazekii* and *R. typhi,* the causative agents of human epidemic typhus and murine typhus, respectively, were reported to express proteins that exhibit phospholipase A2 (PLA2) activity which mobilizes LDs to release fatty acids [104]. *R. prowazekii* protein, RP534 [105], and *R. typhi* proteins, RT0590 and RT0522 [106], are homologous to the *Pseudomonas aeruginosa*, protein ExoU [105,106], which hydrolyzed host cell LDs to produce PGE2. ExoU-mediated PGE2 subsequently prevented *P. aeruginosa* clearance from the host [107,108,109] (reviewed later). *Rickettsial* infection also induced PGE2 production in endothelial cells [110,111], suggesting that RP534, RT0590, and RT0522 might interact with host LDs to potentially produce PGE2. Further investigation is required to determine the existence and importance of LD–*Rickettsia spp.* interaction. Contrary to reports thus far, *O. tsutsugamushi* may not be the only *Rickettsia spp.* that utilizes LDs to facilitate intracellular growth. 

### 2.2. Facultative Intracellular Bacteria

Several pathogens including *Mycobacterium spp.*, *Legionella pneumophila*, *Listeria monocytogenes*, *Burkholderia spp.*, *Francisella tularensis* and *Salmonella spp.* possess the ability to survive and replicate intracellularly as well as extracellularly. Like obligate intracellular bacteria, these pathogens are present extracellularly before invading the host cells, followed by an intracellular phase which provides a shield from the host humoral immune response and facilitates bacterial replication. However, facultative intracellular bacteria possess an additional extracellular phase in the host which represents a particular disease stage. For example, *Salmonella’s* escape from its initial intracellular niche back into the gut lumen occurs during this stage of its infectious cycle [112]. This extracellular phase within the host uses additional virulence factors to produce specific disease pathologies and provides the pathogen with a survival advantage. Classically, sepsis is a common outcome of a facultative intracellular bacterial dissemination [113]. Thus, both the replicative intracellular and disseminative extracellular phase are crucial for pathogenesis [113,114]. Although facultative intracellular bacteria are studied quite extensively, only *Mycobacterium spp.* and *Salmonella* have been reported to interact with host LDs.

#### 2.2.1. *Mycobacterium spp.*

*Mycobacterium spp*. cause diseases of global importance such as tuberculosis and leprosy. *M. tuberculosis*, *M. leprae*, and *M. bovis* infections all characteristically result in the formation of foamy, LD-filled macrophages [115,116,117]. This section focuses on the three *Mycobacterium spp*. and their interaction with LDs. 

##### *M.* *tuberculosis* *(Mtb)*

*Mtb* causes severe pulmonary disease tuberculosis (TB) which has afflicted humans for nearly 70,000 years [118]. Despite increasing availability of antibiotics, TB continues to be one of the top 10 causes of death worldwide and poses a great public health burden [119]. Humans acquire *Mtb* via inhalation; once in the respiratory tract, *Mtb* preferentially infects alveolar macrophages, escapes phagolysosomal fusion, and matures within the phagosome in order to evade host immune response-mediated clearance [120]. The bacterium can persist in the lungs latently for several years without manifesting as clinical TB. In latent TB, infiltration of the alveolar macrophages results in granuloma formation, a structure that includes the infected cells, differentiated macrophages, and LD-laden macrophages, all surrounded by T lymphocytes [81,82]. While the granuloma is capable of limiting *Mtb* growth, it is also hypothesized to provide a niche for bacterial survival [115]. Studies implicate that LDs are an important component of the foamy macrophages that allow for *Mtb* persistence within the host [reviewed in [23,116]]. Although several studies thus far use the non-pathogenic *M. smegmatis* to identify the role of LDs, in this section we only discuss data obtained for the more virulent and most commonly used *Mtb* H37Rv strain. 

*Mtb* manipulates host cell lipid metabolism through various mechanisms to form foamy macrophages. In an in vitro granuloma model, *Mtb* very-long-chain fatty acids, namely oxygenated mycolic acids, triggered the differentiation of human monocyte-derived macrophages into LD-laden foamy macrophages [117]. Conversely, in caseous TB granulomas, LD metabolism genes *plin2* and acyl-CoA synthetase (*acs*) expression were responsible for LD accumulation [121]. *Mtb* infection induced LD formation in murine and human macrophages [117], which could also be visualized in the newly developed in vitro lung alveolar macrophage model using the primary Max Planck Institute (MPI) cells [122]. On a cellular level, *Mtb* dysregulated host lipid metabolism primarily through manipulation of PPAR nuclear receptors to induce host LD accumulation. While the adipogenic ability of PPARγ induced LD formation and thereby bacterial growth [123], PPARα activation however promoted LD catabolism and fatty acid β-oxidation, thus reducing the bacterial load [124]. A similar difference in PPAR family member activity was observed in *M. bovis*-infected cells discussed later. While the activation of transcription factor EB (TFEB) was essential for PPARα activation [124], PPARγ activation relied on vitamin D receptor activity. Vitamin D treatment of *Mtb*-infected macrophages downregulated PPARγ activation, abating the infection-induced LD accumulation and ultimately reducing bacterial growth [123]. Thus, the PPAR family plays an important role in *Mtb*-induced LD accumulation. 

In addition to inducing host cell LDs, *Mtb* also synthesized its own TAG-containing LDs, hypothesized to support the bacterium during dormancy [125,126,127,128]. The fatty acid composition of *Mtb* and host TAG is nearly identical, suggesting that the bacterium utilized its own TAG synthase in conjunction with host TAGs to form bacterial LDs [129]. *Mtb* also preferentially migrated towards host LDs in foamy macrophages, ultimately engulfing these LDs and accumulating the lipids within. This observation suggested that host LDs may serve as a source of nutrients for bacterial long-term persistence [117]. *Mtb* is reported to express a gene cluster *mec4* that codes for a cholesterol import system, supporting the claim that *Mtb* may use lipids, in this case cholesterol, as a carbon source. Although cholesterol was not required for murine infection or growth in macrophages, it appeared to be essential for bacterial persistence within the murine lungs [130]. Further, *Mtb* is reported to code for phospholipase C, an enzyme that catalyzes the release of stored free fatty acids, a carbon and energy source [131]. Therefore, most available studies primarily propose that LDs provide both carbon substrates for replication and an energy source for *Mtb* survival. However, a recent study demonstrated that in resting macrophages devoid of LDs, the bacterium readily accumulated host lipids from other sources [132]. This suggests that LDs are not the primary lipid source during *Mtb* infection. The authors demonstrated that LD formation during *Mtb* infection in vitro and in vivo required IFNγ, a host cytokine important for bacterial control [133]. Hence, LD formation in this model appears to be an immune response which subsequently promoted the production of host protective eicosanoids, suggesting that LDs are predominantly beneficial for the host and not the bacterium. Notably, the *Mtb* strain used in this study, Erdman, is less virulent compared to the H37Rv strain used in all other studies discussed [134], potentially altering the macrophage response. 

Another recent study also proposed that host-induced LD formation contributes to a proinflammatory response during *Mtb* pathogenesis [135]. In the guinea pig model of pulmonary TB, necrotic granulomas exhibited increased proinflammatory TNFα levels directly proportional to TAG accumulation. Similarly, during in vitro *Mtb* infection of human macrophages, host TAG synthesis increased during necrosis, a process that classically upregulates the pro-inflammatory cytokine response. The authors suggested that, using lipase mediated degradation, the macrophages potentially obtain lipids from the necrotic cells and subsequently differentiate into LD-rich foamy macrophages [135]. Thus, LDs formed during necrotic TB could serve as a source of proinflammatory cytokines, thereby exacerbating rather than promoting pathogen survival. Although much is known about *Mtb* and LDs, the intricate host–pathogen interplay indicates a need for further investigation as well as validation of results through more consistent model and strain usage. 

##### *M.* *bovis*

*M. bovis* is the causative agent of bovine tuberculosis and infects humans who consume undercooked meat. It is also the progenitor of the *M. bovis* bacillus Calmette-Guerin (BCG) strain used in the tuberculosis vaccine. *M. bovis* infection induced LD accumulation in murine peritoneal [136] and bone marrow-derived macrophages [124], the newly established *Galleria mellonella* infection model [137], and C57BL/6 mice [138]. In murine infection, *M. bovis* BCG induced early neutrophil recruitment to the site of inflammation followed by neutrophil apoptosis. Phagocytosis of apoptotic neutrophils resulted in LD formation and the subsequent production of the anti-inflammatory mediators PGE2 and TGFβ, which facilitated bacterial survival [138]. In *M. bovis*-infected murine peritoneal macrophages, the increase in LDs and PGE2 production required PPARγ activation via a TLR-2 dependent pathway [136]. Independently, IL-6 promoted *M. bovis* BCG-induced LD formation and bacterial survival [139]. Although PPARγ activation inhibits the production of IL-6 [140], the involvement of TLR2-mediated PPARγ activation in IL-6 production specifically in BCG-infected macrophages should be investigated. Interestingly, PPARα activation in *M. bovis*-infected macrophages induced LD catabolism and promoted an antimicrobial response [124]. Similar to *Mtb*, each PPAR family member exhibits a distinct role during *M. bovis* infection based on the tissue, cell type, experimental conditions. Thus, LDs are essential for *M. bovis* infection, but their contribution to pathogenesis remains elusive. 

##### *M.* *leprae*

Although *M. leprae* appears to have evolved from the common *M. tuberculosis* ancestor, it preferentially invades the Schwann and glial (macrophage) cells of the peripheral nervous system to cause nerve damage and leprosy. Two major manifestations of the disease include tuberculoid leprosy, resulting in granuloma formation, and Schwann cell death, and lepromatous leprosy characterized by LD-rich foam cell-containing lesions. Elamin et al. [30] have extensively reviewed the long-standing LD-*M. leprae* association Rudolf Virchow first described in 1863 [28,30]. LDs were observed in skin [141] and nerve [142] biopsies from lepromatous leprosy patients as well as in vitro in infected murine peritoneal macrophages [141], human PBMC-derived macrophages [141], human macrophage-like THP-1 cell line [143] and Schwann cells [142,144]. Further, *M. leprae* infection induced *plin1* and *plin2* upregulation in murine and human macrophages [141,143] in vitro and in lepromatous leprosy skin biopsies [141]. In THP-1 cells, this upregulation required TLR2 receptor signaling [143]. Usually, the TLR2 ligand peptidoglycan attenuates *plin2* expression. However, in THP-1 cells, *M. leprae* infection appeared to override this blockage, causing LD accumulation [143]. Notably, TLR2 and TLR6 deletion only partially affected LD formation in human and murine peritoneal macrophages, suggesting involvement of alternative receptors [141]. In infected Schwann cells, the deletion of TLR6 and not TLR2, completely abolished LD accumulation. The difference in macrophage and Schwann cell TLR2 dependence could be attributed to their different immune capabilities. In Schwann cells, LD localization to *M. leprae*-containing phagosomes also relied on cytoskeletal rearrangement and PI3K signaling [142]. Infection of Schwann cells with recombinant *M. bovis* BCG that expresses a *M. leprae* phenolic glycolipid I (PGLI), important for bacterial entry into host cell induced expression of a PPARγ-dependent endocytic mannose receptor (MR/CD206) required for LD accumulation. Blocking MR/CD206 decreased bacterial entry and survival suggesting the importance of the mannose receptor in infected Schwann cell LD accumulation [145]. These studies highlight the differences in receptor signaling required for LD accumulation in *M. leprae*-infected macrophages and Schwann cells.

Reduced bacterial growth in Schwann cells [142] and macrophages devoid of LDs demonstrated the importance of LD accumulation for *M. leprae* pathogenesis [30]. However, the specific mechanisms LDs employ to assist *M. leprae* growth have not been elucidated. Besides contributing to the foamy appearance of lepromatous leprosy lesions, LDs provide nutrients and substrates for innate immune response mediators [reviewed extensively in [28]]. Notably, while *M. leprae* infection increased *plin2* expression in macrophages, it downregulated the LD lipolysis enzyme *hsl* to facilitate LD accumulation. Treatment of macrophages with clofazimine, an antibiotic used to treat leprosy, attenuated *plin2* mRNA and protein levels while increasing *hsl* in THP-1 cells and clinical samples. This correlated with reduced LD accumulation and *M. leprae* growth [146], suggesting that HSL inhibition and LD accumulation is important for *M. leprae* survival. 

Bacterial infection significantly increased PGE2 in human and murine macrophages [141] and Schwann cells [147]. Additionally, in Schwann cells, LD biogenesis is correlated with increased PGE2 and IL-10 secretion, as well as reduced IL-12 and NO production, all together, downregulating the immune response [147]. Nerve biopsies from lepromatous leprosy patients demonstrated the colocalization of *M. leprae*, LDs, and COX-2 in Schwann cells, pointing to LDs as possible PGE2 synthesis sites in vivo [147]. Moreover, LD formation and PGE2 production was also induced in Schwann cells infected with the recombinant *M. bovis* BCG expressing the *M. leprae* PGLI. This CD206-PPARγ crosstalk-mediated effect favored intracellular bacterial persistence with concomitant secretion of inflammatory mediators such as IL-8, a possible inducer of neuroinflammation [145]. Thus, in macrophages and Schwann cells, *M. leprae*-induced LDs favor inhibition of bactericidal activities and downregulation of the immune response along with providing nutrients. PGE2 production requires free fatty acids derived from LD breakdown. The *M. leprae* genome codes for two lipases and one phospholipase which could potentially promote LD lipolysis [148]. In addition, overexpression of host lipases and phospholipases in lepromatous leprosy lesions suggested that host enzymes may complement these bacterial genes [149]. Beside free fatty acids for PGE2 production, LDs could also provide nutrients for bacterial growth. However, the time of LD lipolysis post-infection would identify the precise use of LDs. 

Albeit with differences in mechanisms of accumulation, LDs interact with the three mycobacterial species at the interphase of nutrient acquisition and/or the host immune response evasion. Notably, LDs also contribute to foam cell formation and hence the disease pathology of the discussed mycobacterial species infections. 

#### 2.2.2. *Salmonella spp.*

*Salmonella*, a Gram-negative, facultative intracellular enteric bacterial pathogen can infect a variety of hosts including birds, reptiles and mammals. *Salmonella enterica* serovar Typhimurium (*S.* Typhimurium) causes human gastroenteritis and a murine typhoid-like disease associated with systemic infection. The closely related *Salmonella enterica* serovar Typhi (*S.* Typhi) causes human typhoid fever, a frequently fatal systemic disease. Altogether, *Salmonella* causes more than one billion new human infections each year, with a significant mortality rate [150]. Of note, the emergence of multi-drug resistant strains renders *Salmonella spp.* as a major public health concern [151]. *Salmonella spp*. can infect and survive in non-phagocytic cells as well as professional phagocytes, preferentially macrophages [152]. Once inside the cell, the bacterium multiplies in a *Salmonella*-containing vacuole (SCV) and, similar to *C. trachomatis*, employs its Type 3 secretion systems (T3SS) to translocate bacterial effectors into the cell cytoplasm to manipulate host processes [152,153]. The *S. typhimurium* T3SS effector SseJ, with acyltransferase activity [154,155,156,157], assisted in membrane lipid modification and vesicular fusion during SCV biogenesis [157]. Infection of HeLa epithelial cells and the murine RAW 264.7 macrophage cell line with the SseJ mutant resulted in reduced cholesterol esterification and LD accumulation [156], suggesting the importance of LDs during SCV biogenesis. As SseJ is important for SCV membrane dynamics [157], it is thought to esterify cholesterol in order to maintain SCV membrane fluidity, signaling, and vesicular transport. Additionally, reduced virulence of SseJ mutants in murine infection models [157,158,159] suggested that SseJ-mediated host cholesterol esterification and LD accumulation might play an important role in bacterial virulence. Contrary to SseJ, infection with the T3SS effector SseL mutant accumulated large amounts of LDs in the in vivo murine gall bladder infection model, an effect restored with complementation [160]. The SseL-mediated reduction in LD accumulation was dependent on its deubiquitinase activity [160], suggesting that *Salmonella* directly modifies cellular ubiquitination patterns to manipulate host lipid homeostasis. Although, SseL deficiency rendered the bacterium elongated and filamentous, it did not alter bacterial intracellular growth [160]. As the alteration of host lipid metabolism was essential for *Salmonella* infection in the intestinal tract and liver [161], SseL may provide access to the host lipid stores and/or may prevent cellular LD buildup to avoid host inflammatory or stress responses. The two studies discussed provide evidence of *Salmonella*–LD interactions. However, the benefits of their association remain unclear. Although *Salmonella* actively targets host LDs via its T3SS, the role of LDs could be variable depending on the effector and the infection model. 

Intracellular bacteria represent a pathogen group that displays many diverse interactions with LDs. These bacteria manipulate LDs for immune modulation and/or to obtain energy and nutrients required to propagate infection. The diverse mechanisms of LD accumulation and utilization emphasize the common theme of incomplete understanding. Due to the large disease burden of intracellular bacteria, further elucidation of the LD–intracellular pathogen interaction could help explore novel pathways and proteins that could be targeted in future. 

## 3. Extracellular Bacteria

Unlike intracellular bacteria, extracellular bacteria can move within the body and organs but do not invade cells. While they can proliferate in the host extracellular environment, they lack the ability to either facilitate their own cellular uptake or to survive the intracellular environment. Some extracellular bacteria like *Vibrio cholerae* [162] and *Bordetella pertussis* [163] adhere to epithelial surfaces and secrete toxins to cause disease. Alternatively, *Escherichia coli* and *Pseudomonas aeruginosa* spread rapidly to various interstitial spaces in the body after entering the host [164]. Even though extracellular bacteria have a significantly different lifestyle compared to intracellular bacteria, they also utilize host cell lipids during infection [reviewed in [23]]. For example, *B. burgdorferi,* the causative agent of Lyme disease, cannot synthesize cholesterol and requires host cholesterol to support its growth and multiplication [165]. Similarly, *Helicobacter pylori,* the causative agent of chronic atrophic gastritis and stomach and duodenal ulceration, destroys host lipid rafts to liberate host cholesterol [166]. Several extracellular bacteria incorporate host lipids into their membrane to maintain structural integrity. However, the mechanism extracellular bacteria employ to acquire host cell lipids remains elusive. As LDs are a significant source of lipids for intracellular bacteria, it is imperative to discuss LD–extracellular bacterial relationship and identify LD importance. Thus far, only *P. aeruginosa* and *V. cholerae* have shown direct evidence suggesting a potential role of LDs during their pathogenesis. We will also explore the putative LD–*Streptococcus pyogenes* interaction.

### 3.1. Pseudomonas aeruginosa

*P. aeruginosa* is a Gram-negative, rod-shaped, opportunistic pathogen which causes chronic infections in cystic fibrosis patients, diabetics, and burn patients [167]. It is responsible for an estimated 51,000 nosocomial infections in the United States each year. Additionally, the emerging multidrug resistance of *P. aeruginosa* increases the burden to the healthcare industry [168], emphasizing the need to understand its pathogenesis. To successfully invade the host, *P. aeruginosa* utilizes an array of virulence factors including flagella, pili and lipopolysaccharide. The bacterium releases effector proteins via its T3SS to manipulate the host and establish infection [169]. The effector proteins identified so far include ExoS, ExoT, ExoY, and ExoU. While ExoS and ExoT inhibit actin polymerization, prevent phagocytosis, and promote apoptosis [170], ExoY increases cell membrane permeability [171] and ExoU is reported to possess cytosolic PLA2 activity [172,173] and interact with host cell LDs. In infected airway epithelial cells, ExoU PLA2 induced PGE2 production which then interfered with neutrophil migration [174,175], thereby enhancing bacterial survival within the host suggesting the importance of ExoU-dependent PGE2 in *P. aeruginosa* pathogenesis [107,108,109]. Furthermore, when compared to uninfected and ExoU mutant-infected airway epithelial cells, wild-type bacterium-infected cells showed decreased LD numbers, suggesting an ExoU-dependent decrease in cellular LD numbers [176]. This is unlike the increased LD accumulation observed in most intracellular pathogen-infected cells. Additionally, the deleterious effect of blocking PLA2 activity on the bacterium indicated that *P. aeruginosa* infection reduces LD accumulation via the ExoU PLA2 activity [176]. Overall, these studies suggest that *P. aeruginosa* secretes the T3SS protein ExoU in order to hydrolyze host LDs to increase PGE2 levels, thereby preventing neutrophil infiltration and supporting bacterial growth [177]. 

Along with ExoU, a *P. aeruginosa* quorum sensing-associated molecule, *N*-(3-oxododecanoyl)-l-homoserine lactone (AHL-12), also interacted with host cell LDs [178]. AHL-12 activated mammalian cells, induced chemotaxis, up-regulated the leukocyte receptor CD11b expression, and enhanced polymorphonuclear neutrophil phagocytosis [179,180,181]. Interestingly, AHL-12 colocalized with the bitter receptor T2R38 present on neutrophil LD membranes [178], suggesting T2R38 is a receptor for *P. aeruginosa* AHL-12. It was proposed that this interaction may influence neutrophil activation in a way that sustains bacterial pathogenesis [178]. Additionally, because LDs contain several proteins within their phospholipid monolayer [182], they may act as a hub of receptors for bacterial virulence proteins to bind to, a currently unique role of LDs that requires further elucidation. 

### 3.2. Streptococcus pyogenes

*S. pyogenes* is a Gram-positive bacterium that can cause superficial infections (pharyngitis and pyoderma), invasive infections (necrotizing fasciitis and streptococcal toxic shock syndrome), and post-infectious diseases (rheumatic fever) [183]. *S. pyogenes* is reported to express the protein SlaA, which exhibited PLA2-like activity, similar to the *P. aeruginosa* ExoU protein. Although the presence of SlaA increased the severity of *S. pyogenes* infection [184], its mechanism for bolstering bacterial virulence remains unknown. Interestingly, bacterial infection induced upregulation of PGE2 synthesis enzyme cyclooxygenase-2 (*cox-2*) which correlated with increased severity of infection in patients as well as murine experimental models [185,186]. Further, COX-2-mediated PGE2 production suppressed host cell phagocytic capacity and prevented *S. pyogenes* clearance similar to *P. aeruginosa* [186], suggesting an anti-inflammatory role of PGE2. Although SlaA exhibits PLA2 activity similar to *P. aeruginosa* ExoU, whether SlaA contributes to PGE2 production during *S. pyogenes* infection is not known. Based on the known contribution of PLA2 activity exhibiting bacterial virulence factors in LD-derived PGE2 production, the role of LDs in *S. pyogenes* infection is worth investigating.

### 3.3. Vibrio cholerae

Infection with the Gram-negative, facultative anaerobe, *V. cholerae*, results in a potentially life-threatening diarrhea most often seen in the developing world. During infection, the bacterium secretes cholera toxin, which continually stimulates adenylate cyclase within enterocytes, thus resulting in a characteristically profuse, watery diarrhea [187]. Along with the most well-known cholera toxin, the bacterium’s virulence also depends on its ability to acquire fatty acids from the environment and assimilate them into the cell membrane. The bacterium uses these fatty acids to endure the toxic reactive oxygen species it encounters while entering a host and initiating infection. As *V. cholerae* is an extracellular pathogen present in contaminated water, it utilizes the polyunsaturated fatty acids from the aquatic organisms to maintain membrane structural integrity [188]. *Vibrio spp.* possess machinery to accumulate either host or environmental fatty acids in order to alter their fatty acid composition and minimize bacterial membrane disturbances [188,189]. The presence of these protective mechanisms emphasizes the importance of fatty acids in *Vibrio spp.* pathogenesis. However, whether the bacteria manipulate host LDs to acquire fatty acids remains elusive. LDs accumulated in *V. cholerae*-infected *Drosophila melanogaster* [187,190] as well as in human embryonic kidney (HEK 293) cells, both commonly used models to mimic human infection [187]. In infected *Drosophila*, LD accumulation in enterocytes directly correlated with host death [190] and was dependent upon the bacterial two-component system CrbRS (carboxylic acid regulator and sensor). CrbRS activates acetyl-CoA synthase (*acs-1*) transcription to facilitate bacterial transition from host acetate excretion to assimilation [190]. As acetate contributes significantly to host intestinal and systemic health [191], bacterial CrbRS-mediated sequestration of acetate deactivated host insulin signaling, resulting in altered lipid accumulation within the enterocytes, which caused intestinal steatosis and ultimately host death [190]. The accumulated large-sized LDs could be due to TAG accumulation within the enterocyte or LD coalescence to compensate for the limited supply of short chain phospholipids. Although LC–MS/MS lipidomic analysis revealed no change in TAGs in the *V. cholerae*-infected *Drosophila* intestine, it detected a significant decrease in both phosphatidylcholine (PC) and phosphatidylethanolamine (PE) species with total fatty acid carbons totaling less than or equal to 30. This suggested that phospholipid catabolism results in LD coalescence contributing to their large appearance [187]. Whether *V. cholerae* utilizes these short chain fatty acids for bacterial membrane remodeling remains unknown. LD coalescence resulted from *V. cholerae* consumption of dietary methionine sulfoxide (MetO) in the *Drosophila* intestinal lumen [187]. The lack of dietary MetO activated host methionine sulfoxide reductase A (MsrA) to reduce protein-associated MetO and activated host proteins involved in phospholipid degradation; lipid depletion thus caused LD coalescence and ultimately host death. Besides acetate supplementation [190], knockdown of MsrA expression in enterocytes or infection with *V. cholerae* unable to consume dietary MetO increased host survival [187]. When less MsrA is available for host protein repair, the phospholipid degradation cascade is blocked, and the host survives. These studies indicated that *V*. *cholerae* consumption of dietary MetO promotes host protein functions essential for bacterial virulence [187]. Thus, LDs play a unique role during *V. cholerae* infection of *Drosophila*, where the accumulation of large LDs in the enterocytes indicates host death. However, LDs have not been reported in *V. cholerae* human infection. Future studies should investigate whether *V. cholerae* utilizes the catabolized host phospholipids to maintain their membrane structural integrity.

LDs play a distinct role in extracellular bacterial infections compared to intracellular bacteria. Similar to certain intracellular bacteria, *P. aeruginosa* utilizes LDs to induce PGE2 production. However, it reduces cellular LD accumulation via its effector protein, contrary to other intracellular bacteria such as *Chlamydia spp*., *C. burnetii* and *Mycobacterium spp.* Moreover, in *P. aeruginosa*-infected cells, proteins present on LDs serve as potential receptors for bacterial ligands; this role has not yet been studied in intracellular bacterial pathogenesis. Uniquely in *V. cholerae* pathogenesis, LD accumulation is deleterious to the infected host, rather than a gauge of bacterial infectivity observed for several intracellular bacteria. Thus, LDs serve distinct roles during extracellular bacterial pathogenesis compared to intracellular bacteria which could be attributed to the differences in their lifestyles and infection cycle. 

## 4. Gut Microbiota 

The gut microbiota is comprised of several commensal bacteria which maintain energy homeostasis and contribute significantly to nutrient absorption and enterocyte lipid metabolism to maintain overall host health. Thus, while not pathogenic, it is important to discuss gut microbiota influence on the host LD homeostasis. In zebrafish (*Danio rerio*) intestinal epithelium, LD accumulation decreased in germ-free and antibiotic-treated larvae compared to conventional larvae [192,193]. This observation indicated that zebrafish intestinal microbiota has a significant influence on LD accumulation. Furthermore, in *Caenorhabditis elegans,* the foodborne microbiota influenced LD accumulation [194]. *C. elegans* fed with lactic acid bacteria (LAB) obtained from Mozzarella di Bufala Campana (MBC), a traditional Italian cheese, showed higher LD accumulation compared to commercial probiotic strain, *L. rhamnosus* GG (LGG)-fed worms. In contrast to the LGG-fed worms, the *Lactobacillus delbrueckii, L. fermentum*, and *Leuconostoc lactis*-containing LAB displayed larger LDs and reduced C. elegans longevity [194]. This suggests that foodborne microbial consortium impacts host fat metabolism and subsequent LD accumulation. However, the specific contribution of LDs to C. elegans life span remains unknown [194]. Similarly, in mice and cellular models, different commensals influence enterocyte lipid absorption in the small intestine under normal diet conditions. While Escherichia coli colonization was associated with reduced enterocyte LD size, *Lactobacillus paracasei* induced a shift toward larger-sized LDs [195]. It is speculated that *E. coli* colonization may cause the host to derive less energy from carbohydrates, instead utilizing dietary fat absorption and subsequent fatty acid β-oxidation to fuel metabolic processes. Conversely, *L. paracasei* gut colonization could result in a higher level of energy extraction from complex polysaccharides and, consequently, lower level of fat absorption [195]. Therefore, *E. coli* and *L. paracasei* might have distinct effects on lipid metabolism, resulting in enhanced lipid catabolism and increased lipid storage in cytosolic LDs, respectively [195]. As the gut microbiota influences LDs in the intestine, it is important to investigate the gut microbiota–LD relationship at the interphase of host gastrointestinal health maintenance and obesity as well as intestinal pathogen infections.

## 5. Conclusions

**Figure 1:**
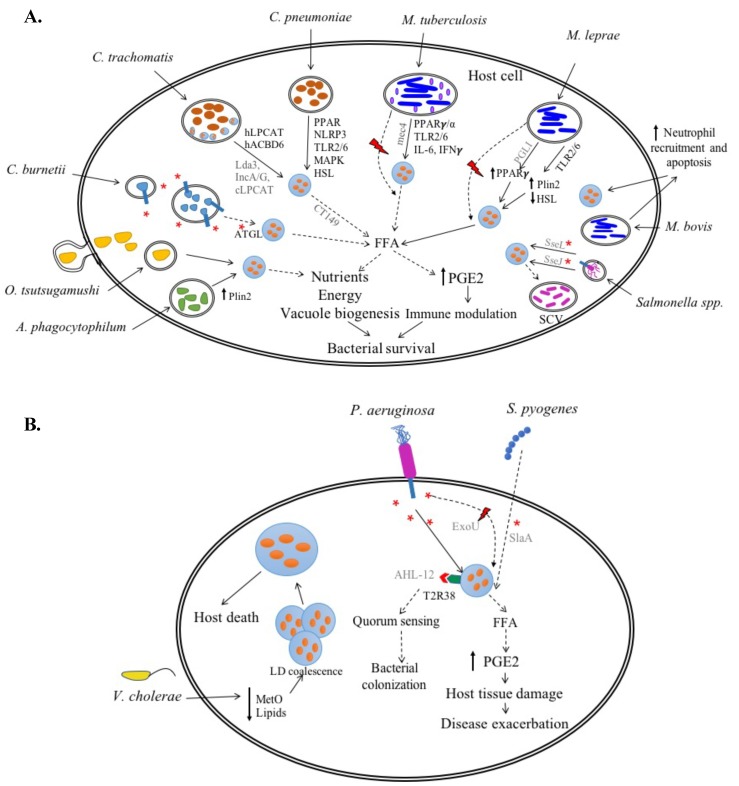
Overview of host LD–bacterial interactions. (**A**) Intracellular bacteria employ different host (black) and bacterial proteins (grey) to induce LD accumulation or LD lipolysis to generate free fatty acids (FFA) as a source of energy, nutrients or prostaglandin E2 (PGE2). However, the function of LDs is still unclear for most pathogens as indicated by the dotted arrows. *Salmonella* is hypothesized to utilize LDs for *Salmonella*-containing vacuole (SCV) biogenesis and host LDs translocate to the *C. tachomatis* vacuole. *Mtb* is the only bacterium known thus far to synthesize bacterial LD. (**B**) Extracellular pathogen *P. aeruginosa* utilizes LDs to either increase PGE2 production or uses receptors on LDs for bacterial protein binding. LD coalescence in *V. cholerae*-infected host enterocyte is an indicator of host death. *S. pyogenes* is hypothesized to target LDs. * secretion system effector, 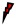
—bacterial phospholipases.

Various bacteria manipulate host lipid metabolism to promote infection [36]. Particularly, the eukaryotic neutral lipid storage organelles, LDs, are emerging as important players of host–pathogen interactions. Both intracellular as well as extracellular bacteria target host LDs for successful infection. However, the inconsistencies in the in vivo and in vitro models and techniques used, and the mere dearth of reported studies has resulted in deficient knowledge regarding host LD–bacterial interactions. Based on the current data, the appreciable differences in bacterial lifestyles seem to allow for unique host LD–intracellular and –extracellular bacterial interactions while also exhibiting some similarities (Figure 1, Table 1). Nonetheless, future investigations should identify the precise mechanisms bacteria use to target host LDs, the host and/or bacterial proteins involved, and the LD contribution to bacterial growth and disease pathology. 

Since Virchow’s first observation of LD-containing foam cells in *M. leprae*-infected patients [28,30], several intracellular bacteria have been reported to increase host cell LD numbers, esterified cellular lipid concentrations or LD coat protein *plin* expression, suggesting association with host LDs. Unlike several intracellular bacteria, *P. aeruginosa* infection reduces host LD accumulation and *V. cholerae* induces LD coalescence, thus exhibiting unique extracellular bacteria–host LD interactions. Despite distinct outcomes, the extracellular *P. aeruginosa* and the intracellular *C. burnetii, C. trachomatis* and *Salmonella spp*. all employ secreted bacterial effector proteins to alter host LDs. However, the exact mechanisms of action and which host proteins the effectors target is not known. For example, *C. burnetii* requires functional bacterial T4SS and host ATGL-mediated LD lipolysis for optimal growth. However, whether the T4SS effectors directly target host cell ATGL requires further investigation. Similarly, *Salmonella spp.* T3SS effector SseJ uses a yet unknown mechanism to target host LDs. Future studies should identify and elucidate the mechanism of action of bacterial proteins that alter host LDs. 

The involvement of bacterial proteins suggests that host LDs are beneficial for the pathogens. While the formation of LDs is important for some bacteria, LD lipolysis is essential for others (Table 1). During latency, *M. leprae* and *Mtb* persist in the granuloma where LDs in the foamy macrophages potentially assist dormant bacteria. Furthermore, the presence of *C. pneumoniae* and *C. burnetii* in foam cells is also associated with atherogenesis and Q fever endocarditis respectively, suggesting LD contribution to disease outcomes and bacterial survival. The importance of LDs to intracellular bacterial growth and disease pathology, and the involvement of bacterial proteins suggests that host LD alteration is a bacterium-driven process. However, other studies suggest that LDs are beneficial for the host. LD coat protein Plin2 and LD-associated immunity-related GTPase Irgm3 assist in dendritic cell cross-presentation to activate a CD8 T cell response [196], important for intracellular bacteria *Listeria monocytogenes* [197], *Mtb* [198] and *Salmonella* [199] clearance. Additionally, bacterial ligands promote the release of LD-associated histones which have an antibacterial effect on Gram-positive *Staphylococcus epidermidis* and Gram-negative *Escherichia coli* [200]. Further, LD accumulation in *Mycobacterium spp.* requires immune-activating bacterial ligands, host pattern recognition receptors and proinflammatory cytokine IFNγ suggesting host cell LD alteration as a host-driven process. Interestingly, *Mtb* and *M. leprae* code for phospholipases which catalyze the release of fatty acids from phospholipid membranes, a potential source of carbon and energy and may be a reciprocal response to evade the host-driven LD accumulation. Determining the bacterial phospholipase expression, the host LD turnover and the effect of LD formation or lipolysis on bacterial growth at various times post-infection will help elucidate LD alteration as a host or a bacterium-driven process.

Irrespective of how LDs are altered, they certainly seem to promote intracellular bacterial growth. LDs are hypothesized to provide nutrients and energy for intracellular survival, lipids for bacterial vacuolar membrane biogenesis, and modulators for immune evasion (Figure 1). *C. pneumoniae*, *M. leprae* and *Mtb*, which reside in foam cells, potentially use host LD-derived free fatty acids to generate ATP via β-oxidation. Whether other intracellular bacteria use LDs as a source of energy should be investigated. Notably, *Mtb* utilizes the host LD-derived TAGs and bacterial TAG synthase to form its own LDs hypothesized to assist the bacterium during dormancy. Whether other bacteria synthesize their own LDs remains unknown and should be explored as a potential bacterial mechanism to utilize the host LDs as a nutrient source. Unlike intracellular bacteria, *V. cholerae* is not yet known to utilize LDs for nutrition. However, bacterial consumption of host cell MetO results in host nutritional imbalance, affects host lipid homeostasis, results in LD coalescence and subsequently host death. The role of LDs as an indicator of *V. cholerae*-infected host death requires further investigation. Besides nutrients, the obligate intracellular bacteria *C. trachomatis*, *C. pneumoniae*, *C. burnetii*, and *A. phagocytophilum* also require host cell lipids and cholesterol to establish respective parasitophorous vacuoles, their intracellular niche. Similar to *Salmonella spp*. which utilizes LD-derived lipids to assist *Salmonella*-containing vacuole (SCV) biogenesis, other intracellular pathogens could utilize LD-derived lipids to assist in vacuolar biogenesis. Although not for parasitophorous vacuole, extracellular *V. cholerae* is hypothesized to use LDs to maintain bacterial membrane structural integrity. Thus, utilization of LD-derived lipids appears to differ based on bacterial lifestyle. 

In *M. leprae*-infected Schwann cells, LDs are associated with increased anti-inflammatory PGE2 and IL-10 and reduced proinflammatory IL-12 and NO secretion resulting in an immunosuppressive environment. Since most intracellular bacteria evade the immune response to survive long-term in the host, bacteria might manipulate host cell LDs to induce anti-inflammatory cytokine secretion. PGE2 contributes to an immunosuppressive environment in *C. pneumoniae*, *C. burnetii* and *M. bovis*-infected hosts, and to host tissue damage and disease exacerbation during *C. trachomatis* and *P. aeruginosa* infection. Since LDs are an active site for eicosanoid synthesis [201], their role as a PGE2 source should be established for the discussed bacteria. Thus, the role of LDs during host–bacteria interactions largely remains unclear. 

Given the vast range of bacterial pathogens, very few have been demonstrated to interact with host LDs, with especially limited information regarding extracellular bacterial pathogens. *Escherichia coli*, *Acinetobacter baumannii*, *Klebsiella pneumonia*, *Proteus vulgaris*, and *Staphylococcus aureus* [20,202] are suggested to interact with host LDs with very little direct evidence. Along with *S. aureus* [203], *Rickettsia spp*. and *S. pyogenes* express bacterial phospholipases similar to *P. aeruginosa, Mtb* and *M. leprae*. Since phospholipases are important for LD lipolysis, which provides free fatty acids as a source of nutrients, energy and precursors for PGE2 production, identifying the interaction of these pathogens with LDs is important. Interestingly, LDs also play an important role in gut microbiota suggesting a potential interaction with intestinal pathogens. Hence, the role of LDs in other enteric pathogens besides the discussed *Salmonella spp.* should be identified. Further, the relation between gut bacteria-mediated LD accumulation and obesity should be explored. 

Besides identifying new bacteria–LD interactions, better understanding of how the currently reported bacteria associate with LDs is essential. Although LDs play an important role in bacterial pathogenesis, even after so many years of research, the findings discussed here only scratch the surface of LD–bacterial interaction. Several questions still remain unanswered; (1) Are there significant differences in LD interactions between intracellular and extracellular bacteria? (2) Is LD alteration a host-driven, a bacterium-driven or a dynamic phenomenon? (3) Which host and bacterial proteins are involved and what is their mechanism of action? and (4) At what stage of bacterial growth are LDs important? Answering these questions for each bacterium will provide critical insight into the importance of LDs during bacterial infections, thus elucidating a novel host–bacterial pathogen interaction pathway. 

## Figures and Tables

**Table 1:** Effect of lipid droplets (LDs) on bacterial growth.

**Obligate Intracellular Bacteria (Vacuolar)**	
*Chlamydia trachomatis*	LD formation required for optimal growth
*Chlamydia pneumoniae*	LD formation required for growth
*Coxiella burnetii*	LD breakdown essential for growth
*Anaplasma phagocytophilum*	LD formation increases growth
**Obligate Intracellular Bacteria (Cytoplasmic)**	
*Orientia tsutsugamushi*	LD formation increases growth
**Facultative Intracellular Bacteria**	
*Mycobacterium tuberculosis*	Contrasting roles
*Mycobacterium bovis*	Contrasting roles
*Mycobacterium leprae*	LD formation essential for growth
*Salmonella spp.*	Unknown
**Extracellular Bacteria**	
*Pseudomonas aeruginosa*	Unknown
*Vibrio cholerae*	Unknown

## References

[1] Tauchi-Sato K., Ozeki S., Houjou T., Taguchi R., Fujimoto T. (2002). The surface of lipid droplets is a phospholipid monolayer with a unique Fatty Acid composition. J. Biol. Chem..

[2] Martin S., Parton R.G. (2006). Lipid droplets: A unified view of a dynamic organelle. Nat. Rev. Mol. Cell Biol..

[3] Pol A., Gross S.P., Parton R.G. (2014). Review: Biogenesis of the multifunctional lipid droplet: Lipids, proteins, and sites. J. Cell Biol..

[4] Cermelli S., Guo Y., Gross S.P., Welte M.A. (2006). The lipid-droplet proteome reveals that droplets are a protein-storage depot. Curr. Biol..

[5] Itabe H., Yamaguchi T., Nimura S., Sasabe N. (2017). Perilipins: A diversity of intracellular lipid droplet proteins. Lipids Health Dis..

[6] Haemmerle G., Lass A., Zimmermann R., Gorkiewicz G., Meyer C., Rozman J., Heldmaier G., Maier R., Theussl C., Eder S. (2006). Defective lipolysis and altered energy metabolism in mice lacking adipose triglyceride lipase. Science.

[7] Vaughan M., Berger J.E., Steinberg D. (1964). Hormone-Sensitive Lipase and Monoglyceride Lipase Activities in Adipose Tissue. J. Biol. Chem..

[8] Guijas C., Rodriguez J.P., Rubio J.M., Balboa M.A., Balsinde J. (2014). Phospholipase A2 regulation of lipid droplet formation. Biochim. Biophys. Acta.

[9] Walther T.C., Farese R.V. (2012). Lipid droplets and cellular lipid metabolism. Ann. Rev. Biochem..

[10] Krahmer N., Farese R.V., Walther T.C. (2013). Balancing the fat: Lipid droplets and human disease. EMBO Mol. Med..

[11] Petan T., Jarc E., Jusovic M. (2018). Lipid Droplets in Cancer: Guardians of Fat in a Stressful World. Molecules.

[12] Pennetta G., Welte M.A. (2018). Emerging Links between Lipid Droplets and Motor Neuron Diseases. Dev. Cell.

[13] Cabirol-Pol M.J., Khalil B., Rival T., Faivre-Sarrailh C., Besson M.T. (2018). Glial lipid droplets and neurodegeneration in a Drosophila model of complex I deficiency. Glia.

[14] Melo R.C., Weller P.F. (2016). Lipid droplets in leukocytes: Organelles linked to inflammatory responses. Exp. Cell Res..

[15] Den Brok M.H., Raaijmakers T.K., Collado-Camps E., Adema G.J. (2018). Lipid Droplets as Immune Modulators in Myeloid Cells. Trends Immunol..

[16] Herker E., Ott M. (2012). Emerging role of lipid droplets in host/pathogen interactions. J. Biol. Chem..

[17] Roingeard P., Melo R.C. (2017). Lipid droplet hijacking by intracellular pathogens. Cell. Microbiol..

[18] Saka H.A., Valdivia R. (2012). Emerging roles for lipid droplets in immunity and host-pathogen interactions. Ann. Rev. Cell Dev. Biol..

[19] Sorgi C.A., Secatto A., Fontanari C., Turato W.M., Belanger C., de Medeiros A.I., Kashima S., Marleau S., Covas D.T., Bozza P.T. (2009). *Histoplasma capsulatum* cell wall {beta}-glucan induces lipid body formation through CD18, TLR2, and dectin-1 receptors: Correlation with leukotriene B4 generation and role in HIV-1 infection. J. Immunol..

[20] Melo R.C., Dvorak A.M. (2012). Lipid body-phagosome interaction in macrophages during infectious diseases: Host defense or pathogen survival strategy?. PLoS Pathogens.

[21] Samanta D., Mulye M., Clemente T.M., Justis A.V., Gilk S.D. (2017). Manipulation of Host Cholesterol by Obligate Intracellular Bacteria. Front. Cell. Infect. Microbiol..

[22] Stehr M., Elamin A.A., Singh M. (2012). Cytosolic lipid inclusions formed during infection by viral and bacterial pathogens. Microbes Infect. Institut. Pasteur..

[23] Toledo A., Benach J.L. (2015). Hijacking and use of host lipids by intracellular pathogens. Microbiol. Spectr..

[24] Vallochi A.L., Teixeira L., Oliveira K.D.S., Maya-Monteiro C.M., Bozza P.T. (2018). Lipid Droplet, a Key Player in Host-Parasite Interactions. Front. Immunol..

[25] Lavie M., Dubuisson J. (2017). Interplay between hepatitis C virus and lipid metabolism during virus entry and assembly. Biochimistry.

[26] Herker E., Ott M. (2011). Unique ties between hepatitis C virus replication and intracellular lipids. Trends Endocrinol. Metab..

[27] Zhang J., Lan Y., Sanyal S. (2017). Modulation of Lipid Droplet Metabolism-A Potential Target for Therapeutic Intervention in Flaviviridae Infections. Front. Microbiol..

[28] De Mattos K.A., Sarno E.N., Pessolani M.C., Bozza P.T. (2012). Deciphering the contribution of lipid droplets in leprosy: Multifunctional organelles with roles in Mycobacterium leprae pathogenesis. Memórias do Instituto Oswaldo Cruz.

[29] Barisch C., Soldati T. (2017). Breaking fat! How mycobacteria and other intracellular pathogens manipulate host lipid droplets. Biochimie.

[30] Elamin A.A., Stehr M., Singh M. (2012). Lipid Droplets and *Mycobacterium leprae* Infection. J. Pathog..

[31] Suter E. (1956). Interaction between phagocytes and pathogenic microorganisms. Bacteriol. Rev..

[32] Moulder J.W. (1985). Comparative biology of intracellular parasitism. Microbiol. Rev..

[33] Raupach B., Kaufmann S.H. (2001). Immune responses to intracellular bacteria. Curr. Opin. Immunol..

[34] Serbina N.V., Pamer E.G. (2008). Coordinating innate immune cells to optimize microbial killing. Immunity.

[35] Knodler L.A., Celli J. (2011). Eating the strangers within: Host control of intracellular bacteria via xenophagy. Cell. Microbiol..

[36] Walpole G.F.W., Grinstein S., Westman J. (2018). The role of lipids in host-pathogen interactions. IUBMB Life.

[37] Becker Y., Baron S. (1996). Chlamydia. Medical Microbiology.

[38] Bastidas R.J., Elwell C.A., Engel J.N., Valdivia R.H. (2013). Chlamydial intracellular survival strategies. Cold Spring Harb. Perspect. Med..

[39] Kumar Y., Cocchiaro J., Valdivia R.H. (2006). The obligate intracellular pathogen *Chlamydia trachomatis* targets host lipid droplets. Curr. Biol..

[40] Saka H.A., Thompson J.W., Chen Y.S., Dubois L.G., Haas J.T., Moseley A., Valdivia R.H. (2015). *Chlamydia trachomatis* Infection Leads to Defined Alterations to the Lipid Droplet Proteome in Epithelial Cells. PLoS ONE.

[41] Sharma M., Recuero-Checa M.A., Fan F.Y., Dean D. (2018). *Chlamydia trachomatis* regulates growth and development in response to host cell fatty acid availability in the absence of lipid droplets. Cell. Microbiol..

[42] Cocchiaro J.L., Kumar Y., Fischer E.R., Hackstadt T., Valdivia R.H. (2008). Cytoplasmic lipid droplets are translocated into the lumen of the *Chlamydia trachomatis* parasitophorous vacuole. Proc. Natl. Acad. Sci. USA.

[43] Rank R.G., Whittimore J., Bowlin A.K., Wyrick P.B. (2011). In vivo ultrastructural analysis of the intimate relationship between polymorphonuclear leukocytes and the chlamydial developmental cycle. Infect. Immun..

[44] Hackstadt T., Scidmore-Carlson M.A., Shaw E.I., Fischer E.R. (1999). The *Chlamydia trachomatis* IncA protein is required for homotypic vesicle fusion. Cell. Microbiol..

[45] Recuero-Checa M.A., Sharma M., Lau C., Watkins P.A., Gaydos C.A., Dean D. (2016). *Chlamydia trachomatis* growth and development requires the activity of host Long-chain Acyl-CoA Synthetases (ACSLs). Sci. Rep..

[46] Peters J., Byrne G.I. (2015). *Chlamydia trachomatis* growth depends on eukaryotic cholesterol esterification and is affected by Acyl-CoA:cholesterol acyltransferase inhibition. Pathog. Dis..

[47] Soupene E., Kuypers F.A. (2017). Phosphatidylserine decarboxylase CT699, lysophospholipid acyltransferase CT775, and acyl-ACP synthase CT776 provide membrane lipid diversity to *Chlamydia trachomatis*. Sci. Rep..

[48] Soupene E., Rothschild J., Kuypers F.A., Dean D. (2012). Eukaryotic protein recruitment into the *Chlamydia* inclusion: Implications for survival and growth. PLoS ONE.

[49] Peters J., Onguri V., Nishimoto S.K., Marion T.N., Byrne G.I. (2012). The *Chlamydia trachomatis* CT149 protein exhibits esterase activity in vitro and catalyzes cholesteryl ester hydrolysis when expressed in HeLa cells. Microbes Infect..

[50] Soupene E., Wang D., Kuypers F.A. (2015). Remodeling of host phosphatidylcholine by Chlamydia acyltransferase is regulated by acyl-CoA binding protein ACBD6 associated with lipid droplets. Microbiologyopen.

[51] Fukuda E.Y., Lad S.P., Mikolon D.P., Iacobelli-Martinez M., Li E. (2005). Activation of lipid metabolism contributes to interleukin-8 production during *Chlamydia trachomatis* infection of cervical epithelial cells. Infect. Immun..

[52] Belland R.J., Ouellette S.P., Gieffers J., Byrne G.I. (2004). *Chlamydia pneumoniae* and atherosclerosis. Cell. Microbiol..

[53] Campbell L.A., Kuo C.C. (2004). Chlamydia pneumoniae—An infectious risk factor for atherosclerosis?. Nat. Rev. Microbiol..

[54] Mori M., Itabe H., Higashi Y., Fujimoto Y., Shiomi M., Yoshizumi M., Ouch Y., Takano T. (2001). Foam cell formation containing lipid droplets enriched with free cholesterol by hyperlipidemic serum. J. Lipid Res..

[55] Bobryshev Y.V., Killingsworth M.C., Tran D., Lord R. (2008). Amalgamation of *Chlamydia pneumoniae* inclusions with lipid droplets in foam cells in human atherosclerotic plaque. Virchows Arch..

[56] Cheng B., Wu X., Sun S., Wu Q., Mei C., Xu Q., Wu J., He P. (2014). MAPK-PPARalpha/gamma signal transduction pathways are involved in *Chlamydia pneumoniae*-induced macrophage-derived foam cell formation. Microb. Pathog..

[57] He P., Mei C., Cheng B., Liu W., Wang Y., Wan J. (2009). *Chlamydia pneumoniae* induces macrophage-derived foam cell formation by up-regulating acyl-coenzyme A: Cholesterol acyltransferase 1. Microbes Infect..

[58] Mei C.L., He P., Cheng B., Liu W., Wang Y.F., Wan J.J. (2009). *Chlamydia pneumoniae* induces macrophage-derived foam cell formation via PPAR alpha and PPAR gamma-dependent pathways. Cell Biol. Int..

[59] Walenna N.F., Kurihara Y., Chou B., Ishii K., Soejima T., Itoh R., Shimizu A., Ichinohe T., Hiromatsu K. (2018). *Chlamydia pneumoniae* exploits adipocyte lipid chaperone FABP4 to facilitate fat mobilization and intracellular growth in murine adipocytes. Biochem. Biophys. Res. Commun..

[60] Barbier O., Torra I.P., Duguay Y., Blanquart C., Fruchart J.C., Glineur C., Staels B. (2002). Pleiotropic actions of peroxisome proliferator-activated receptors in lipid metabolism and atherosclerosis. Arterioscler. Thromb. Vasc. Biol..

[61] Lee C.H., Evans R.M. (2002). Peroxisome proliferator-activated receptor-gamma in macrophage lipid homeostasis. Trends Endocrinol. Metab..

[62] Castrillo A., Tontonoz P. (2004). Nuclear receptors in macrophage biology: At the crossroads of lipid metabolism and inflammation. Annu. Rev. Cell Dev. Biol..

[63] Chawla A., Boisvert W.A., Lee C.H., Laffitte B.A., Barak Y., Joseph S.B., Liao D., Nagy L., Edwards P.A., Curtiss L.K. (2001). A PPAR gamma-LXR-ABCA1 pathway in macrophages is involved in cholesterol efflux and atherogenesis. Mol. Cell.

[64] Mei S., Gu H., Ward A., Yang X., Guo H., He K., Liu Z., Cao W. (2012). p38 mitogen-activated protein kinase (MAPK) promotes cholesterol ester accumulation in macrophages through inhibition of macroautophagy. J. Biol. Chem..

[65] Itoh R., Murakami I., Chou B., Ishii K., Soejima T., Suzuki T., Hiromatsu K. (2014). Chlamydia pneumoniae harness host NLRP3 inflammasome-mediated caspase-1 activation for optimal intracellular growth in murine macrophages. Biochem. Biophys. Res. Commun..

[66] Cao F., Castrillo A., Tontonoz P., Re F., Byrne G.I. (2007). *Chlamydia pneumoniae*—Induced macrophage foam cell formation is mediated by Toll-like receptor 2. Infect. Immun..

[67] Rupp J., Berger M., Reiling N., Gieffers J., Lindschau C., Haller H., Dalhoff K., Maass M. (2004). Cox-2 inhibition abrogates *Chlamydia pneumoniae*-induced PGE2 and MMP-1 expression. Biochem. Biophys. Res. Commun..

[68] Van Schaik E.J., Chen C., Mertens K., Weber M.M., Samuel J.E. (2013). Molecular pathogenesis of the obligate intracellular bacterium *Coxiella burnetii*. Nat. Rev. Microbiol..

[69] Voth D.E., Heinzen R.A. (2007). Lounging in a lysosome: The intracellular lifestyle of *Coxiella burnetii*. Cell. Microbiol..

[70] Beare P.A., Gilk S.D., Larson C.L., Hill J., Stead C.M., Omsland A., Cockrell D.C., Howe D., Voth D.E., Heinzen R.A. (2011). Dot/Icm type IVB secretion system requirements for *Coxiella burnetii* growth in human macrophages. MBio.

[71] De Matteis M.A., Godi A. (2004). PI-loting membrane traffic. Nat. Cell Biol..

[72] Howe D., Heinzen R.A. (2006). *Coxiella burnetii* inhabits a cholesterol-rich vacuole and influences cellular cholesterol metabolism. Cell. Microbiol..

[73] Gilk S.D., Cockrell D.C., Luterbach C., Hansen B., Knodler L.A., Ibarra J.A., Steele-Mortimer O., Heinzen R.A. (2013). Bacterial colonization of host cells in the absence of cholesterol. PLoS Pathog..

[74] Beare P.A., Unsworth N., Andoh M., Voth D.E., Omsland A., Gilk S.D., Williams K.P., Sobral B.W., Kupko J.J., Porcella S.F. (2009). Comparative genomics reveal extensive transposon-mediated genomic plasticity and diversity among potential effector proteins within the genus Coxiella. Infect. Immun..

[75] Mulye M., Samanta D., Winfree S., Heinzen R.A., Gilk S.D. (2017). Elevated Cholesterol in the *Coxiella burnetii* Intracellular Niche Is Bacteriolytic. MBio.

[76] Mahapatra S., Ayoubi P., Shaw E.I. (2010). *Coxiella burnetii* Nine Mile II proteins modulate gene expression of monocytic host cells during infection. BMC Microbiol..

[77] Ren Q., Robertson S.J., Howe D., Barrows L.F., Heinzen R.A. (2003). Comparative DNA microarray analysis of host cell transcriptional responses to infection by *Coxiella burnetii* or *Chlamydia trachomatis*. Ann. N. Y. Acad. Sci..

[78] Mulye M., Zapata B., Gilk S.D. (2018). Altering lipid droplet homeostasis affects Coxiella burnetii intracellular growth. PLoS ONE.

[79] Brouqui P., Dumler J.S., Raoult D. (1994). Immunohistologic demonstration of *Coxiella burnetii* in the valves of patients with Q fever endocarditis. Am. J. Med..

[80] Graham J.G., MacDonald L.J., Hussain S.K., Sharma U.M., Kurten R.C., Voth D.E. (2013). Virulent *Coxiella burnetii* pathotypes productively infect primary human alveolar macrophages. Cell. Microbiol..

[81] Sandoz K.M., Valiant W.G., Eriksen S.G., Hruby D.E., Allen R.D., Rockey D.D. (2014). The broad-spectrum antiviral compound ST-669 restricts chlamydial inclusion development and bacterial growth and localizes to host cell lipid droplets within treated cells. Antimicrob. Agents Chemother..

[82] Stead C.M., Cockrell D.C., Beare P.A., Miller H.E., Heinzen R.A. (2018). A *Coxiella burnetii* phospholipase A homolog pldA is required for optimal growth in macrophages and developmental form lipid remodeling. BMC Microbiol..

[83] Koster F.T., Williams J.C., Goodwin J.S. (1985). Cellular immunity in Q fever: Modulation of responsiveness by a suppressor T cell-monocyte circuit. J. Immunol..

[84] Shannon J.G., Heinzen R.A. (2009). Adaptive immunity to the obligate intracellular pathogen *Coxiella burnetii*. Immunol Res..

[85] Izzo A.A., Marmion B.P. (1993). Variation in interferon-gamma responses to *Coxiella burnetii* antigens with lymphocytes from vaccinated or naturally infected subjects. Clin. Exp. Immunol..

[86] Murray P.R., Rosenthal K.S., Pfaller M.A., Philadelphia P.A. (2016). Medical Microbiology.

[87] Dumler J.S., Barbet A.F., Bekker C.P., Dasch G.A., Palmer G.H., Ray S.C., Rikihisa Y., Rurangirwa F.R. (2001). Reorganization of genera in the families *Rickettsiaceae* and *Anaplasmataceae* in the order *Rickettsiales*: Unification of some species of *Ehrlichia* with *Anaplasma*, *Cowdria* with *Ehrlichia* and *Ehrlichia* with *Neorickettsia*, descriptions of six new species combinations and designation of *Ehrlichia equi* and ’HGE agent’ as subjective synonyms of *Ehrlichia phagocytophila*. Int. J. Syst. Evol. Microbiol..

[88] Lin M., Rikihisa Y. (2003). Obligatory intracellular parasitism by *Ehrlichia chaffeensis* and *Anaplasma phagocytophilum* involves caveolae and glycosylphosphatidylinositol-anchored proteins. Cell. Microbiol..

[89] Xiong Q., Lin M., Rikihisa Y. (2009). Cholesterol-dependent *Anaplasma phagocytophilum* exploits the low-density lipoprotein uptake pathway. PLoS Pathog..

[90] Lin M., Rikihisa Y. (2003). *Ehrlichia chaffeensis* and *Anaplasma phagocytophilum* lack genes for lipid A biosynthesis and incorporate cholesterol for their survival. Infect. Immun..

[91] De la Fuente J., Ayoubi P., Blouin E.F., Almazan C., Naranjo V., Kocan K.M. (2005). Gene expression profiling of human promyelocytic cells in response to infection with *Anaplasma phagocytophilum*. Cell. Microbiol..

[92] Manzano-Roman R., Almazan C., Naranjo V., Blouin E.F., Kocan K.M., de la Fuente J. (2008). Expression of perilipin in human promyelocytic cells in response to *Anaplasma phagocytophilum* infection results in modified lipid metabolism. J. Med. Microbiol..

[93] Niu H., Kozjak-Pavlovic V., Rudel T., Rikihisa Y. (2010). *Anaplasma phagocytophilum* Ats-1 is imported into host cell mitochondria and interferes with apoptosis induction. PLoS Pathog..

[94] Beyer A.R., Truchan H.K., May L.J., Walker N.J., Borjesson D.L., Carlyon J.A. (2015). The *Anaplasma phagocytophilum* effector AmpA hijacks host cell SUMOylation. Cell. Microbiol..

[95] Moore H.P., Silver R.B., Mottillo E.P., Bernlohr D.A., Granneman J.G. (2005). Perilipin targets a novel pool of lipid droplets for lipolytic attack by hormone-sensitive lipase. J. Biol. Chem..

[96] Miyoshi H., Souza S.C., Zhang H.H., Strissel K.J., Christoffolete M.A., Kovsan J., Rudich A., Kraemer F.B., Bianco A.C., Obin M.S. (2006). Perilipin promotes hormone-sensitive lipase-mediated adipocyte lipolysis via phosphorylation-dependent and -independent mechanisms. J. Biol. Chem..

[97] Rikihisa Y. (2010). Anaplasma phagocytophilum and Ehrlichia chaffeensis: Subversive manipulators of host cells. Nat. Rev. Microbiol..

[98] McPherson R.A., Pincus M.R. (2017). Henry’s Clinical Diagnosis and Management by Laboratory Methods.

[99] Seong S.Y., Choi M.S., Kim I.S. (2001). *Orientia tsutsugamushi* infection: Overview and immune responses. Microbes Infect..

[100] Chu H., Lee J.H., Han S.H., Kim S.Y., Cho N.H., Kim I.S., Choi M.S. (2006). Exploitation of the endocytic pathway by *Orientia tsutsugamushi* in nonprofessional phagocytes. Infect. Immun..

[101] Ewing E.P., Takeuchi A., Shirai A., Osterman J.V. (1978). Experimental infection of mouse peritoneal mesothelium with scrub typhus rickettsiae: An ultrastructural study. Infect. Immun..

[102] Kim M.J., Kim M.K., Kang J.S. (2013). Involvement of lipid rafts in the budding-like exit of *Orientia tsutsugamushi*. Microb. Pathog..

[103] Ogawa M., Fukasawa M., Satoh M., Hanada K., Saijo M., Uchiyama T., Ando S. (2014). The intracellular pathogen *Orientia tsutsugamushi* responsible for scrub typhus induces lipid droplet formation in mouse fibroblasts. Microbes Infect..

[104] Dennis E.A. (2015). Introduction to Thematic Review Series: Phospholipases: Central Role in Lipid Signaling and Disease. J. Lipid Res..

[105] Housley N.A., Winkler H.H., Audia J.P. (2011). The *Rickettsia prowazekii* ExoU homologue possesses phospholipase A1 (PLA1), PLA2, and lyso-PLA2 activities and can function in the absence of any eukaryotic cofactors in vitro. J. Bacteriol..

[106] Rahman M.S., Gillespie J.J., Kaur S.J., Sears K.T., Ceraul S.M., Beier-Sexton M., Azad A.F. (2013). *Rickettsia typhi* possesses phospholipase A2 enzymes that are involved in infection of host cells. PLoS Pathog..

[107] Guillemot L., Medina M., Pernet E., Leduc D., Chignard M., Touqui L., Wu Y. (2014). Cytosolic phospholipase A2alpha enhances mouse mortality induced by *Pseudomonas aeruginosa* pulmonary infection via interleukin 6. Biochimie.

[108] Hurley B.P., Pirzai W., Mumy K.L., Gronert K., McCormick B.A. (2011). Selective eicosanoid-generating capacity of cytoplasmic phospholipase A2 in *Pseudomonas aeruginosa*-infected epithelial cells. Am. J. Physiol. Lung Cell Mol. Physiol..

[109] Saliba A.M., Nascimento D.O., Silva M.C., Assis M.C., Gayer C.R., Raymond B., Coelho M.G., Marques E.A., Touqui L., Albano R.M. (2005). Eicosanoid-mediated proinflammatory activity of *Pseudomonas aeruginosa* ExoU. Cell. Microbiol..

[110] Rydkina E., Sahni A., Baggs R.B., Silverman D.J., Sahni S.K. (2006). Infection of human endothelial cells with spotted Fever group rickettsiae stimulates cyclooxygenase 2 expression and release of vasoactive prostaglandins. Infect. Immun..

[111] Walker T.S., Brown J.S., Hoover C.S., Morgan D.A. (1990). Endothelial prostaglandin secretion: Effects of typhus rickettsiae. J. Infect. Dis..

[112] Knodler L.A., Vallance B.A., Celli J., Winfree S., Hansen B., Montero M., Steele-Mortimera O. (2010). Dissemination of invasive *Salmonella* via bacterial-induced extrusion of mucosal epithelia. Proc. Natl. Acad. Sci. USA.

[113] Silva M.T. (2012). Classical labeling of bacterial pathogens according to their lifestyle in the host: Inconsistencies and alternatives. Front. Microbiol..

[114] Casadevall A. (2008). Evolution of intracellular pathogens. Annu. Rev. Microbiol..

[115] Ehlers S., Schaible U.E. (2012). The granuloma in tuberculosis: Dynamics of a host-pathogen collusion. Front. Immunol..

[116] Almeida P.E., Carneiro A.B., Silva A.R., Bozza P.T. (2012). PPARgamma Expression and Function in Mycobacterial Infection: Roles in Lipid Metabolism, Immunity, and Bacterial Killing. PPAR Res..

[117] Peyron P., Vaubourgeix J., Poquet Y., Levillain F., Botanch C., Bardou F., Daffé M., Emile J.F., Marchou B., Cardona P.J. (2008). Foamy macrophages from tuberculous patients’ granulomas constitute a nutrient-rich reservoir for *M. tuberculosis* persistence. PLoS Pathog..

[118] Zumla A., Chakaya J., Centis R., D’Ambrosio L., Mwaba P., Bates M., Kapata N., Nyirenda T., Chanda D., Mfinanga S. (2015). Tuberculosis treatment and management—An update on treatment regimens, trials, new drugs, and adjunct therapies. Lancet Respir. Med..

[119] World Health Organization (2017). Global Tuberculosis Report 2017.

[120] Weber S.S., Ragaz C., Hilbi H. (2009). Pathogen trafficking pathways and host phosphoinositide metabolism. Mol. Microbiol..

[121] Kim M.J., Wainwright H.C., Locketz M., Bekker L.G., Walther G.B., Dittrich C., Visser A., Wang W., Hsu F.F., Wiehart U. (2010). Caseation of human tuberculosis granulomas correlates with elevated host lipid metabolism. EMBO Mol. Med..

[122] Woo M., Wood C., Kwon D., Park K.P., Fejer G., Delorme V. (2018). *Mycobacterium tuberculosis* Infection and Innate Responses in a New Model of Lung Alveolar Macrophages. Front. Immunol..

[123] Salamon H., Bruiners N., Lakehal K., Shi L., Ravi J., Yamaguchi K.D., Gennaro M.L. (2014). Cutting edge: Vitamin D regulates lipid metabolism in *Mycobacterium tuberculosis* infection. J. Immunol..

[124] Kim Y.S., Lee H.M., Kim J.K., Yang C.S., Kim T.S., Jung M., Jin H.S., Kim S., Jang J., Oh G.T. (2017). PPAR-alpha Activation Mediates Innate Host Defense through Induction of TFEB and Lipid Catabolism. J. Immunol..

[125] Daniel J., Sirakova T., Kolattukudy P. (2014). An acyl-CoA synthetase in *Mycobacterium tuberculosis* involved in triacylglycerol accumulation during dormancy. PLoS ONE.

[126] Daniel J., Kapoor N., Sirakova T., Sinha R., Kolattukudy P. (2016). The perilipin-like PPE15 protein in *Mycobacterium tuberculosis* is required for triacylglycerol accumulation under dormancy-inducing conditions. Mol. Microbiol..

[127] Elamin A.A., Stehr M., Spallek R., Rohde M., Singh M. (2011). The *Mycobacterium tuberculosis* Ag85A is a novel diacylglycerol acyltransferase involved in lipid body formation. Mol. Microbiol..

[128] Armstrong R.M., Adams K.L., Zilisch J.E., Bretl D.J., Sato H., Anderson D.M., Zahrt T.C. (2016). Rv2744c Is a PspA Ortholog That Regulates Lipid Droplet Homeostasis and Nonreplicating Persistence in *Mycobacterium tuberculosis*. J. Bacteriol..

[129] Daniel J., Maamar H., Deb C., Sirakova T.D., Kolattukudy P.E. (2011). *Mycobacterium tuberculosis* uses host triacylglycerol to accumulate lipid droplets and acquires a dormancy-like phenotype in lipid-loaded macrophages. PLoS Pathog..

[130] Pandey A.K., Sassetti C.M. (2008). *Mycobacterial* persistence requires the utilization of host cholesterol. Proc. Natl. Acad. Sci. USA.

[131] Cole S.T., Brosch R., Parkhill J., Garnier T., Churcher C., Harris D., Gordon S.V., Eiglmeier K., Gas S., Barry C.E. (1998). Deciphering the biology of *Mycobacterium tuberculosis* from the complete genome sequence. Nature.

[132] Knight M., Braverman J., Asfaha K., Gronert K., Stanley S. (2018). Lipid droplet formation in *Mycobacterium tuberculosis* infected macrophages requires IFN-gamma/HIF-1alpha signaling and supports host defense. PLoS Pathog..

[133] Flynn J.L., Chan J., Triebold K.J., Dalton D.K., Stewart T.A., Bloom B.R. (1993). An essential role for interferon gamma in resistance to *Mycobacterium tuberculosis* infection. J. Exp. Med..

[134] Dunn P.L., North R.J. (1995). Virulence ranking of some *Mycobacterium tuberculosis* and *Mycobacterium bovis* strains according to their ability to multiply in the lungs, induce lung pathology, and cause mortality in mice. Infect. Immun..

[135] Jaisinghani N., Dawa S., Singh K., Nandy A., Menon D., Bhandari P.D., Khare G., Tyagi A., Gandotra S. (2018). Necrosis Driven Triglyceride Synthesis Primes Macrophages for Inflammation During *Mycobacterium tuberculosis* Infection. Front. Immunol..

[136] Almeida P.E., Silva A.R., Maya-Monteiro C.M., Torocsik D., D’Avila H., Dezso B., Magalhães K.G., Castro-Faria-Neto H.C., Nagy L., Bozza P.T. (2009). *Mycobacterium bovis* bacillus Calmette-Guerin infection induces TLR2-dependent peroxisome proliferator-activated receptor gamma expression and activation: Functions in inflammation, lipid metabolism, and pathogenesis. J. Immunol..

[137] Li Y., Spiropoulos J., Cooley W., Khara J.S., Gladstone C.A., Asai M., Bossé J.T., Robertson B.D., Newton S.M., Langford P.R. (2018). *Galleria mellonella*—A novel infection model for the *Mycobacterium tuberculosis* complex. Virulence.

[138] D’Avila H., Melo R.C., Parreira G.G., Werneck-Barroso E., Castro-Faria-Neto H.C., Bozza P.T. (2006). *Mycobacterium bovis* bacillus Calmette-Guerin induces TLR2-mediated formation of lipid bodies: Intracellular domains for eicosanoid synthesis in vivo. J. Immunol..

[139] Pean C.B., Schiebler M., Tan S.W., Sharrock J.A., Kierdorf K., Brown K.P., Maserumule M.C., Menezes S., Pilátová M., Bronda K. (2017). Regulation of phagocyte triglyceride by a STAT-ATG2 pathway controls mycobacterial infection. Nat. Commun..

[140] Wang D., Shi L., Xin W., Xu J., Xu J., Li Q., Xu Z., Wang J., Wang G., Yao W. (2017). Activation of PPARgamma inhibits pro-inflammatory cytokines production by upregulation of miR-124 in vitro and in vivo. Biochem. Biophys. Res. Commun..

[141] Mattos K.A., D’Avila H., Rodrigues L.S., Oliveira V.G., Sarno E.N., Atella G.C., Pereira G.M., Bozza P.T., Pessolani M.C. (2010). Lipid droplet formation in leprosy: Toll-like receptor-regulated organelles involved in eicosanoid formation and *Mycobacterium leprae* pathogenesis. J. Leukoc. Biol..

[142] Mattos K.A., Lara F.A., Oliveira V.G., Rodrigues L.S., D’Avila H., Melo R.C., Manso P.P., Sarno E.N., Bozza P.T., Pessolani M.C. (2011). Modulation of lipid droplets by *Mycobacterium leprae* in Schwann cells: A putative mechanism for host lipid acquisition and bacterial survival in phagosomes. Cell. Microbiol..

[143] Tanigawa K., Suzuki K., Nakamura K., Akama T., Kawashima A., Wu H., Hayashi M., Takahashi S., Ikuyama S., Ito T. (2008). Expression of adipose differentiation-related protein (ADRP) and perilipin in macrophages infected with Mycobacterium leprae. FEMS Microbiol. Lett..

[144] Jin S.H., An S.K., Lee S.B. (2017). The formation of lipid droplets favors intracellular *Mycobacterium leprae* survival in SW-10, non-myelinating Schwann cells. PLoS Negl. Trop. Dis..

[145] Diaz Acosta C.C., Dias A.A., Rosa T., Batista-Silva L.R., Rosa P.S., Toledo-Pinto T.G., Costa F.D.M.R., Lara F.A., Rodrigues L.S., Mattos K.A. (2018). PGL I expression in live bacteria allows activation of a CD206/PPARgamma cross-talk that may contribute to successful *Mycobacterium leprae* colonization of peripheral nerves. PLoS Pathog..

[146] Degang Y., Akama T., Hara T., Tanigawa K., Ishido Y., Gidoh M., Makino M., Ishii N., Suzuki K. (2012). Clofazimine modulates the expression of lipid metabolism proteins in *Mycobacterium leprae*-infected macrophages. PLoS Negl. Trop. Dis..

[147] Mattos K.A., Oliveira V.G., D’Avila H., Rodrigues L.S., Pinheiro R.O., Sarno E.N., Pessolani M.C., Bozza P.T. (2011). TLR6-driven lipid droplets in *Mycobacterium leprae*-infected Schwann cells: Immunoinflammatory platforms associated with bacterial persistence. J. Immunol..

[148] Cruz D., Watson A.D., Miller C.S., Montoya D., Ochoa M.T., Sieling P.A., Gutierrez M.A., Navab M., Reddy S.T., Witztum J.L. (2008). Host-derived oxidized phospholipids and HDL regulate innate immunity in human leprosy. J. Clin. Invest..

[149] Cole S.T., Eiglmeier K., Parkhill J., James K.D., Thomson N.R., Wheeler P.R., Honoré N., Garnier T., Churcher C., Harris D. (2001). Massive gene decay in the leprosy bacillus. Nature.

[150] Pang T., Bhutta Z.A., Finlay B.B., Altwegg M. (1995). Typhoid fever and other salmonellosis: A continuing challenge. Trends Microbiol..

[151] Boyle E.C., Bishop J.L., Grassl G.A., Finlay B.B. (2007). *Salmonella*: From pathogenesis to therapeutics. J. Bacteriol..

[152] Ohl M.E., Miller S.I. (2001). *Salmonella*: A model for bacterial pathogenesis. Annu. Rev. Med..

[153] Cossart P., Sansonetti P.J. (2004). Bacterial invasion: The paradigms of enteroinvasive pathogens. Science.

[154] Buckley J.T. (1982). Substrate specificity of bacterial glycerophospholipid:cholesterol acyltransferase. Biochemistry.

[155] MacIntyre S., Buckley J.T. (1978). Presence of glycerophospholipid: Cholesterol acyltransferase and phospholipase in culture supernatant of *Aeromonas hydrophila*. J. Bacteriol..

[156] Nawabi P., Catron D.M., Haldar K. (2008). Esterification of cholesterol by a type III secretion effector during intracellular Salmonella infection. Mol. Microbiol..

[157] Ruiz-Albert J., Yu X.J., Beuzon C.R., Blakey A.N., Galyov E.E., Holden D.W. (2002). Complementary activities of SseJ and SifA regulate dynamics of the *Salmonella typhimurium* vacuolar membrane. Mol. Microbiol..

[158] Lawley T.D., Chan K., Thompson L.J., Kim C.C., Govoni G.R., Monack D.M. (2006). Genome-wide screen for *Salmonella* genes required for long-term systemic infection of the mouse. PLoS Pathog..

[159] Freeman J.A., Ohl M.E., Miller S.I. (2003). The Salmonella enterica serovar typhimurium translocated effectors SseJ and SifB are targeted to the Salmonella-containing vacuole. Infect. Immun..

[160] Arena E.T., Auweter S.D., Antunes L.C., Vogl A.W., Han J., Guttman J.A., Croxen M.A., Menendez A., Covey S.D., Borchers C.H. (2011). The deubiquitinase activity of the *Salmonella* pathogenicity island 2 effector, SseL, prevents accumulation of cellular lipid droplets. Infect. Immun..

[161] Antunes L.C., Andersen S.K., Menendez A., Arena E.T., Han J., Ferreira R.B., Borchers C.H., Finlay B.B. (2011). Metabolomics reveals phospholipids as important nutrient sources during *Salmonella* growth in bile in vitro and in vivo. J. Bacteriol..

[162] Conner J.G., Teschler J.K., Jones C.J., Yildiz F.H. (2016). Staying Alive: *Vibrio cholerae’s* Cycle of Environmental Survival, Transmission, and Dissemination. Microbiol Spectr..

[163] Carbonetti N.H. (2016). *Bordetella pertussis*: New concepts in pathogenesis and treatment. Curr. Opin. Infect. Dis..

[164] Peterson J.W., Baron S. (1996). Bacterial Pathogenesis. Medical Microbiology.

[165] Ostberg Y., Berg S., Comstedt P., Wieslander A., Bergstrom S. (2007). Functional analysis of a lipid galactosyltransferase synthesizing the major envelope lipid in the Lyme disease spirochete *Borrelia burgdorferi*. FEMS Microbiol. Lett..

[166] Wunder C., Churin Y., Winau F., Warnecke D., Vieth M., Lindner B., Zähringer U., Mollenkopf H.J., Heinz E., Meyer T.F. (2006). Cholesterol glucosylation promotes immune evasion by *Helicobacter pylori*. Nat. Med..

[167] Sarabhai S.K.A., Capalash N., Sharma P., Kahlon R. (2016). Quorum Sensing in *Pseudomonas aeruginosa*: Mechanism and Regulation of Virulence. Pseudomonas: Molecular and Applied Biology.

[168] Cohen T.P.D., Prince A., Ramos J.L., Goldberg J. (2015). Pseudomonas aeruginosa Host Immune Evasion.

[169] D’Agata E. (2015). Pseudomonas aeruginosa and Other Pseudomonas Species. Mandell, Douglas, and Bennett’s Principles and Practice of Infectious Diseases.

[170] Barbieri J.T., Sun J. (2004). *Pseudomonas aeruginosa* ExoS and ExoT. Rev. Physiol. Biochem. Pharmacol..

[171] Ochoa C.D., Alexeyev M., Pastukh V., Balczon R., Stevens T. (2012). *Pseudomonas aeruginosa* exotoxin Y is a promiscuous cyclase that increases endothelial tau phosphorylation and permeability. J. Bacteriol..

[172] Deng Q., Barbieri J.T. (2008). Molecular mechanisms of the cytotoxicity of ADP-ribosylating toxins. Annu. Rev. Microbiol..

[173] Anderson D.M., Frank D.W. (2012). Five mechanisms of manipulation by bacterial effectors: A ubiquitous theme. PLoS Pathog..

[174] Sadikot R.T., Zeng H., Azim A.C., Joo M., Dey S.K., Breyer R.M., Peebles R.S., Blackwell T.S., Christman J.W. (2007). Bacterial clearance of *Pseudomonas aeruginosa* is enhanced by the inhibition of COX-2. Eur. J. Immunol..

[175] Aronoff D.M., Bergin I.L., Lewis C., Goel D., O’Brien E., Peters-Golden M., Mancuso P. (2012). E-prostanoid 2 receptor signaling suppresses lung innate immunity against *Streptococcus pneumoniae*. Prostaglandins Other Lipid Mediat..

[176] Plotkowski M.C., Brandao B.A., de Assis M.C., Feliciano L.F., Raymond B., Freitas C., Saliba A.M., Zahm J.M., Touqui L., Bozza P.T. (2008). Lipid body mobilization in the ExoU-induced release of inflammatory mediators by airway epithelial cells. Microb. Pathog..

[177] Phillips R.M., Six D.A., Dennis E.A., Ghosh P. (2003). In vivo phospholipase activity of the Pseudomonas aeruginosa cytotoxin ExoU and protection of mammalian cells with phospholipase A2 inhibitors. J. Biol. Chem..

[178] Maurer S., Wabnitz G.H., Kahle N.A., Stegmaier S., Prior B., Giese T., Gaida M.M., Samstag Y., Hänsch G.M. (2015). Tasting *Pseudomonas aeruginosa* Biofilms: Human Neutrophils Express the Bitter Receptor T2R38 as Sensor for the Quorum Sensing Molecule N-(3-Oxododecanoyl)-l-Homoserine Lactone. Front. Immunol..

[179] Vikstrom E., Magnusson K.E., Pivoriunas A. (2005). The *Pseudomonas aeruginosa* quorum-sensing molecule N-(3-oxododecanoyl)-L-homoserine lactone stimulates phagocytic activity in human macrophages through the p38 MAPK pathway. Microbes Infect..

[180] Zimmermann S., Wagner C., Muller W., Brenner-Weiss G., Hug F., Prior B., Obst U., Hänsch G.M. (2006). Induction of neutrophil chemotaxis by the quorum-sensing molecule N-(3-oxododecanoyl)-L-homoserine lactone. Infect. Immun..

[181] Wagner C., Zimmermann S., Brenner-Weiss G., Hug F., Prior B., Obst U., Hänsch G.M. (2007). The quorum-sensing molecule N-3-oxododecanoyl homoserine lactone (3OC12-HSL) enhances the host defence by activating human polymorphonuclear neutrophils (PMN). Anal. Bioanal. Chem..

[182] Welte M.A. (2007). Proteins under new management: Lipid droplets deliver. Trends Cell Biol..

[183] Cunningham M.W. (2000). Pathogenesis of group A *streptococcal* infections. Clin. Microbiol. Rev..

[184] Sitkiewicz I., Nagiec M.J., Sumby P., Butler S.D., Cywes-Bentley C., Musser J.M. (2006). Emergence of a bacterial clone with enhanced virulence by acquisition of a phage encoding a secreted phospholipase A2. Proc. Natl. Acad. Sci. USA.

[185] Blaschke U., Beineke A., Klemens J., Medina E., Goldmann O. (2017). Induction of Cyclooxygenase 2 by *Streptococcus pyogenes* Is Mediated by Cytolysins. J. Innate Immun..

[186] Goldmann O., Hertzen E., Hecht A., Schmidt H., Lehne S., Norrby-Teglund A., Medina E. (2010). Inducible cyclooxygenase released prostaglandin E2 modulates the severity of infection caused by *Streptococcus pyogenes*. J. Immunol..

[187] Vanhove A.S., Hang S., Vijayakumar V., Wong A.C., Asara J.M., Watnick P.I. (2017). *Vibrio cholerae* ensures function of host proteins required for virulence through consumption of luminal methionine sulfoxide. PLoS Pathog..

[188] Moravec A.R., Siv A.W., Hobby C.R., Lindsay E.N., Norbash L.V., Shults D.J., Symes S.J.K., Giles D.K. (2017). Exogenous Polyunsaturated Fatty Acids Impact Membrane Remodeling and Affect Virulence Phenotypes among Pathogenic Vibrio Species. Appl. Environ. Microbiol..

[189] Giles D.K., Hankins J.V., Guan Z., Trent M.S. (2011). Remodelling of the Vibrio cholerae membrane by incorporation of exogenous fatty acids from host and aquatic environments. Mol. Microbiol..

[190] Hang S., Purdy A.E., Robins W.P., Wang Z., Mandal M., Chang S., Mekalanos J.J., Watnick P.I. (2014). The acetate switch of an intestinal pathogen disrupts host insulin signaling and lipid metabolism. Cell Host Microbe.

[191] Russell W.R., Hoyles L., Flint H.J., Dumas M.E. (2013). Colonic bacterial metabolites and human health. Curr. Opin. Microbiol..

[192] Sheng Y., Ren H., Limbu S.M., Sun Y., Qiao F., Zhai W., Du Z.-Y., Zhang M. (2018). The Presence or Absence of Intestinal Microbiota Affects Lipid Deposition and Related Genes Expression in Zebrafish (*Danio rerio*). Front. Microbiol..

[193] Semova I., Carten J.D., Stombaugh J., Mackey L.C., Knight R., Farber S.A., Rawls J.F. (2012). Microbiota regulate intestinal absorption and metabolism of fatty acids in the zebrafish. Cell Host Microbe.

[194] Zanni E., Laudenzi C., Schifano E., Palleschi C., Perozzi G., Uccelletti D., Devirgiliis C. (2015). Impact of a Complex Food Microbiota on Energy Metabolism in the Model Organism *Caenorhabditis elegans*. Biomed. Res. Int..

[195] Tazi A., Araujo J.R., Mulet C., Arena E.T., Nigro G., Pedron T., Sansonetti P.J. (2018). Disentangling Host-Microbiota Regulation of Lipid Secretion by Enterocytes: Insights from Commensals *Lactobacillus paracasei* and *Escherichia coli*. MBio.

[196] Bougneres L., Helft J., Tiwari S., Vargas P., Chang B.H., Chan L., Campisi L., Lauvau G., Hugues S., Kumar P. (2009). A role for lipid bodies in the cross-presentation of phagocytosed antigens by MHC class I in dendritic cells. Immunity.

[197] Reinicke A.T., Omilusik K.D., Basha G., Jefferies W.A. (2009). Dendritic cell cross-priming is essential for immune responses to *Listeria monocytogenes*. PLoS ONE.

[198] Winau F., Weber S., Sad S., de Diego J., Hoops S.L., Breiden B., Sandhoff K., Brinkmann V., Kaufmann S.H., Schaible U.E. (2006). Apoptotic vesicles crossprime CD8 T cells and protect against tuberculosis. Immunity.

[199] Yrlid U., Wick M.J. (2000). Salmonella-induced apoptosis of infected macrophages results in presentation of a bacteria-encoded antigen after uptake by bystander dendritic cells. J. Exp. Med..

[200] Anand P., Cermelli S., Li Z., Kassan A., Bosch M., Sigua R., Huang L., Ouellette A.J., Pol A., Welte M.A. (2012). A novel role for lipid droplets in the organismal antibacterial response. Elife.

[201] Bozza P.T., Bakker-Abreu I., Navarro-Xavier R.A., Bandeira-Melo C. (2011). Lipid body function in eicosanoid synthesis: An update. Prostaglandins Leukot Essent Fatty Acids.

[202] Nicolaou G., Goodall A.H., Erridge C. (2012). Diverse bacteria promote macrophage foam cell formation via Toll-like receptor-dependent lipid body biosynthesis. J. Atheroscler. Thromb..

[203] Chen X., Alonzo F. (2019). Bacterial lipolysis of immune-activating ligands promotes evasion of innate defenses. Proc. Natl. Acad. Sci. USA.

